# Sepsis biomarkers: recent advances and future perspectives

**DOI:** 10.3389/fimmu.2026.1734588

**Published:** 2026-03-19

**Authors:** Xu Zheng, Hongwu Guo, Jinyu Zhang, Ming Lei, Shuli Chou, Yingfang Liu, Liang Luo

**Affiliations:** 1 Intensive Care Unit, The Seventh Affiliated Hospital, Sun Yat-sen University, Shenzhen, China; 2 Sun Yat-sen University, Guangzhou, China; 3 KeyLaboratory for Systems Medicine in Inflammatory Diseases, Sun Yat-sen University, Shenzhen, China

**Keywords:** biomarkers, early diagnosis, inflammatory factors, metabolic markers, sepsis

## Abstract

Sepsis remains a leading global cause of morbidity and mortality among the critically ill, driven by a dysregulated host response to infection that culminates in systemic inflammation and multi-organ dysfunction. While numerous potential biomarkers have been identified, their translation into robust clinical tools remains challenging. This review synthesizes the current understanding of sepsis biomarkers, focusing on their utility in delineating the intricate pathophysiological mechanisms underlying this heterogeneous syndrome and predicting patient outcomes. Crucially, we emphasize the pivotal role of cutting-edge methodologies, including advanced multi-omics integration, sophisticated bioinformatics, and machine learning algorithms, in accelerating the discovery and validation of novel precision-guided strategies. Through this synthesis, we outline recent advances and remaining knowledge gaps, aiming to inform precision medicine frameworks by highlighting how innovative technologies are reshaping the approach to biomarker identification and clinical application.

## 1 Introduction

Sepsis, a life-threatening condition caused by a dysregulated host response to infection, carries high mortality rates due to resultant multi-organ dysfunction. Current diagnostic paradigms heavily rely on clinical assessment and time-consuming microbiological culture, methods often hampered by delays and insufficient specificity (1). This diagnostic gap can critically impede timely intervention, thereby worsening patient outcomes. Consequently, the identification and validation of robust biomarkers have become a central focus in sepsis research, holding significant promise for enhancing diagnostic accuracy and speed, ultimately refining therapeutic strategies.

Emerging research has delineated multiple molecular candidates with diagnostic and prognostic potential in sepsis. Established inflammatory biomarkers including C-reactive protein (CRP), procalcitonin (PCT), and interleukin-6 (IL-6) exhibit variable diagnostic performance across clinical contexts. Particularly promising are emerging biomarkers such as neutrophil CD64 (nCD64) expression profiles and metabolomic signatures, which may enable dynamic monitoring of disease progression (2, 3). Beyond diagnostic utility, these biomarkers facilitate risk stratification through quantitative biological profiling, permitting personalized therapeutic escalation in high-risk cohorts (4–7).

Despite these advancements, substantial implementation barriers persist. Current limitations include heterogeneous biomarker interpretation guidelines, interpopulation variability in biomarker kinetics, and absence of standardized operational protocols (6, 8). An emerging, potentially transformative strategy involves the integration of biomarkers using machine learning algorithms (9). Throughout this review, biomarkers are discussed across a spectrum of evidence ranging from guideline-endorsed clinical tools to exploratory and preclinical candidates, and their interpretation should reflect the underlying level of validation, we aim to accelerate the transition from biomarker discovery to clinically impactful applications.

## 2 Overview of sepsis: pathophysiology and clinical relevance

Sepsis represents a complex and dynamic pathophysiological continuum, arising from maladaptive host responses to infection. These responses often initiate with systemic inflammatory response syndrome (SIRS) and can progressively escalate into multi-organ dysfunction syndrome (MODS), which is a primary driver of the high morbidity and mortality associated with sepsis (10, 11). The clinical relevance of sepsis is underscored by its status as a leading cause of critical illness globally, demanding prompt recognition and intervention to mitigate adverse outcomes. Understanding the intricate interplay between the host immune system and invading pathogens is crucial for unraveling the mechanisms underlying sepsis progression and identifying potential therapeutic targets. Although broad-spectrum antibiotics and supportive care remain the cornerstones of current management paradigms, the profound heterogeneity observed in sepsis presentations unequivocally necessitates the development of more precise diagnostic and prognostic tools.

Indeed, recent investigations not only illuminate the potential of novel biomarkers across diverse cohorts, but have substantiated their expanding utility across the sepsis continuum—from risk prognostication to therapeutic guidance. For instance, Wang et al.’s multicenter retrospective analysis demonstrated that elevated TyG index significantly predicted sepsis risk in COPD exacerbations (adjusted OR 2.01, 95% CI 1.34–3.02), with concurrent associations for acute kidney injury (OR 1.78, 1.25–2.54), indicating its utility as a metabolic predictor in COPD-associated sepsis (12). Getsina et al. quantified metabolic derangements in pediatric oncology patients through a prospective observational study (n=71: 40 malignancy patients, 31 septic complications). Presepsin demonstrated superior diagnostic accuracy (AUC 0.89, 95% CI 0.82-0.95) compared to procalcitonin (AUC 0.76, 0.66-0.85) in differentiating sepsis from non-infectious inflammation. Persistent metabolic dysregulation independent of inflammatory status (*p* < 0.01) underscores the necessity for multimodal monitoring in this vulnerable population (13). Liu et al. characterized *Providencia stuartii* pathogenesis in non-human primates through comprehensive necropsy analyses. Combined histopathological scoring (Grade III meningoencephalitis) with 16S rRNA sequencing confirmed systemic dissemination, demonstrating neutrophil-to-lymphocyte ratios exceeding 15:1 and CSF bacterial loads >10^5 CFU/mL. This zoonotic model provides critical insights into opportunistic pathogen behavior in immunocompromised hosts (14). Morley et al. implemented a pragmatic RCT (n=1,200) evaluating time-sensitive fluid resuscitation in sepsis. Protocolized intervention within 3h of recognition reduced 28-day mortality (18.2% vs 26.7%; RR 0.68, 0.57-0.82) and mitigated organ failure progression (SOFA score Δ -2.3 vs -1.1; *p* < 0.001). These findings support the importance of early recognition and timely initiation of resuscitative measures, although rigid early goal-directed therapy protocols have not demonstrated consistent survival benefit in contemporary randomized trials (15). Petel et al. conducted a cross-sectional survey of 58 Level IV NICUs, revealing substantial practice variation in late-onset sepsis management. Vancomycin administration in 78% of empirical regimens exceeded pathogen prevalence data (*Methicillin-resistant Staphylococcus aureus* (MRSA) incidence <15%), with only 32% of units adhering to antimicrobial stewardship protocols (16). These findings emphasize the critical need for evidence-based neonatal sepsis guidelines.

## 3 Standard of care and point-of-care biomarkers

### 3.1 Importance of lactate in sepsis

As the terminal product of anaerobic glycolysis, lactate has established itself as an indispensable metabolic biomarker for sepsis management. Hyperlactatemia, particularly lactate concentrations ≥4 mmol/L, is strongly associated with circulatory failure and adverse outcomes in sepsis and is incorporated into Surviving Sepsis Campaign algorithms for risk stratification and resuscitation guidance (17). Lactate’s prognostic value is evidenced by its dose-dependent association with disease severity (adjusted HR 1.32 per 1 mmol/L increase, 95% CI 1.15-1.52) and 28-day mortality in septic cohorts. Persistent hyperlactatemia (>4 mmol/L at 6h post-resuscitation) independently predicts hospital mortality (OR 3.45, 95% CI 2.11-5.63), serving as a stronger prognostic indicator than baseline values. A multicenter cohort study (n=1,432) revealed that lactate clearance <10% at 6h combined with SOFA score ≥9 predicts 90-day mortality with 78% sensitivity (AUC 0.81, *p* < 0.001), outperforming APACHE II in dynamic risk stratification (18).

Current SSC guidelines recommend serial lactate measurements (0, 3, 6h) combined with qSOFA scoring for early shock recognition. Lactate-guided resuscitation strategies have been associated with improved risk stratification and treatment monitoring and have demonstrated non-inferiority to alternative perfusion-guided approaches in randomized trials when interpreted alongside clinical indicators of tissue perfusion. Lactate kinetics provide dynamic feedback on the response to resuscitation. Failure to achieve meaningful lactate clearance within the first hours has been associated with worse outcomes and may prompt reassessment of perfusion and resuscitation strategy in conjunction with clinical findings. Early lactate normalization (<2 mmol/L within 24h) correlates with 58% lower 28-day mortality risk (NNT = 4), whereas sustained levels >4 mmol/L at 12h predict refractory shock with 92% specificity, mandating advanced hemodynamic monitoring (19). These findings position lactate not merely as a diagnostic adjunct but as a cornerstone of goal-directed therapy in critical care.

### 3.2 CRP and PCT

CRP and PCT are key biomarkers in sepsis management, but they differ in specificity and clinical use. CRP is a nonspecific marker that rises in response to inflammation, making it useful for detecting systemic inflammatory conditions. However, its lack of specificity means it can be elevated in various non-infectious conditions, limiting its ability to distinguish sepsis from other inflammatory disorders (20).

In contrast, PCT is more specific to bacterial infections and has become central to guiding antibiotic therapy in sepsis. PCT levels rise sharply during bacterial infections, and its dynamic changes help determine the initiation or de-escalation of antibiotics. Unlike CRP, PCT is less affected by viral infections, making it more reliable for distinguishing bacterial sepsis. Studies have shown that PCT-guided therapy reduces antibiotic duration and improves patient outcomes, particularly in critical care (21, 22).

However, like CRP, PCT levels may also rise in other conditions. Thus, PCT is more valuable for ruling out sepsis than for diagnosing it, and the combination of these two biomarkers can enhance diagnostic performance (23–25).

### 3.3 Presepsin

Presepsin, a fragment of soluble CD14, is an emerging biomarker gaining increasing attention in sepsis diagnosis. Studies have confirmed its strong prognostic value in predicting mortality and organ failure, particularly in emergency department and intensive care unit settings. Presepsin has demonstrated promising diagnostic and prognostic performance in selected cohorts, although its diagnostic accuracy relative to procalcitonin and C-reactive protein remains population-dependent (13, 26, 27).

### 3.4 Mid-regional pro-adrenomedullin

MR-proADM is another promising biomarker for sepsis, particularly in risk stratification. Elevated levels of MR-proADM have been consistently associated with poor outcomes, including higher mortality and organ failure. Across observational cohorts, higher MR-proADM levels are consistently associated with adverse outcomes and may provide incremental prognostic information when combined with established biomarkers (28, 29).However, reported cutoffs and performance metrics are population-dependent, and prospective studies are needed to define how MR-proADM should be integrated into clinical decision pathways.

### 3.5 Standard of care and point-of-care biomarkers

The turnaround time (TAT) and bedside availability of point-of-care (POC) biomarkers are critical for timely clinical decision-making in sepsis management. Biomarkers such as lactate, CRP, and PCT can often be measured within an hour, enabling early intervention. Newer markers like Presepsin and MR-proADM also offer rapid testing, with results typically available within an hour of sample collection. This rapid availability is essential for initiating prompt therapeutic strategies, as delays in treatment can lead to significantly worse outcomes. The integration of these POC biomarkers into routine clinical practice is vital for improving sepsis outcomes. They enable the early identification of septic patients, the rapid initiation of appropriate therapies, and continuous monitoring of treatment efficacy.

## 4 Advancements in sepsis diagnosis: biomarker utilization

Recent innovations in sepsis diagnostics have leveraged multidimensional biomarker strategies through integrated omics approaches. These approaches hold significant promise for improving the accuracy of early diagnosis and precision treatment.

### 4.1 Experimental biomarkers

For instance, Chen et al. (30). adopted untargeted LC-MS/MS metabolomic profiling with Q-Exactive Plus mass spectrometer and RNA sequencing in 16 sepsis vs 11 SIRS patients, identifying 485 differentially expressed genes (FDR<0.05) and 1,083 altered metabolites (VIP>1.5). Notably, four macrophage-associated genes (ITGAM [3.2-fold], CD44 [2.8-fold], C3AR1 [4.1-fold], IL2RG [2.5-fold]) demonstrated differential expression, with functional validation via siRNA knockdown reducing IL-6 and TNF-α secretion by 40-55% (*p* < 0.01), suggesting therapeutic potential for macrophage-targeted strategies.

### 4.2 Clinically validated biomarkers

In neurocritical care, Peng et al. (31). developed a longitudinal prognostic model through linear mixed-effects analysis of 214 patients. Key predictors included Charlson comorbidity index (*β* = 0.32), hemoglobin decline (Δ=-2.1g/dL; *p* = 0.01), and body cell mass/phase angle ratio (BCM/PA; *β* = -0.56; *p* = 0.003). The composite index achieved superior discrimination (AUC 0.95, 0.91-0.98) with BCM/PA ≤ 3.2 kg/° yielding 89% sensitivity for 30-day mortality prediction, demonstrating critical value in time-sensitive assessments. Hasibuan et al. (32). evaluated NLR and PLR in neonatal sepsis (n=137 confirmed cases). NLR demonstrated modest diagnostic accuracy (AUC 0.62, 95% CI 0.54-0.70) with 52.1% sensitivity/50.6% specificity at cutoff 3.2, while PLR showed poorer performance (AUC 0.58, 0.50-0.66). Importantly, integration with clotting time parameters increased accuracy (AUC 0.76), suggesting the necessity for multimodal diagnostic panels combining these indices with established biomarkers. These findings highlight the potential diagnostic value of NLR and PLR in specific populations, including neonatal and pediatric cohorts, although their performance in adult sepsis varies across settings. Fang et al. (33). developed a coagulation index (CI) for septic DIC prediction in 287 patients. Combining prolonged APTT (>45s), platelet count <100×10^9^/L, and fibrinogen <1.5g/L, CI≥3.2 predicted DIC development (OR 5.6, 3.2-9.8) with 84% accuracy, enabling 48-hour earlier detection versus International Society Onthrombosis & Haemostasis (ISTH) criteria (*p* < 0.001). Serial monitoring demonstrated decreasing CI correlated with survival improvement (*β* = 0.42; *p* = 0.008). Keller et al. (34). investigated serum calprotectin in COVID-19 sepsis (n=120). ELISA analysis revealed 2.8-fold elevations in bacterial/fungal superinfections (median 4,560 vs 1,620 ng/mL; *p* < 0.001). At cutoff >3,800 ng/mL, calprotectin displayed 96% specificity (91-99%) and 60% sensitivity (48-71%) for VRE detection (AUC 0.82), outperforming CRP (ΔAUC=0.14; *p* = 0.02), indicating utility in pandemic-associated sepsis triage. Garbern et al. (35). piloted wireless monitoring in pediatric sepsis (n=45). The XGBoost algorithm integrating continuous heart rate (>140bpm), respiration rate (>90th percentile), and perfusion index (<1.2) achieved 2.5h median lead-time (IQR 1.8-3.2) for septic shock prediction (AUROC 0.88, 0.82-0.93). Device implementation in resource-limited settings reduced unwitnessed deterioration events by 62% (*p* = 0.005).

## 5 Specific biomarkers in sepsis: mechanisms and prognostic values

### 5.1 Inflammatory Factors as Biomarkers

#### 5.1.1 The Role of IL-6, IL-10, and TNF-α

Interleukin-6 (IL-6) is a pleiotropic pro-inflammatory cytokine pathologically elevated in critical conditions. As a key mediator of innate immunity, it orchestrates inflammatory cascades and stimulates hepatic acute-phase protein synthesis. Elevated serum IL-6 levels correlate with disease severity, and recent meta-analyses confirm its independent predictive capacity for 28-day mortality (36).Song et al. (37). established IL-6 as a high-performance biomarker. Higher IL-6 concentrations, particularly when persistently elevated, have been independently associated with increased disease severity and mortality, although prognostic thresholds vary across studies (38–40).

In contrast, IL-10 serves as a critical immunoregulatory cytokine, mediating feedback inhibition of pro-inflammatory pathways. Its role in sepsis is paradoxical: while early elevation may indicate a compensatory response, sustained levels predict immunosuppression and secondary infections (41). Tumor necrosis factor-alpha (TNF-α), a prototypic pro-inflammatory cytokine, is closely associated with endothelial activation and capillary leakage. The dynamic equilibrium between pro- and anti-inflammatory mediators determines clinical trajectories, with cytokine ratios (e.g., IL-6/IL-10) often emerging as superior prognostic indicators compared to individual biomarkers (42).

#### 5.1.2 The relationship between dynamic changes in inflammatory factors and prognosis

Dynamic fluctuations in inflammatory mediators including interleukin-6, interleukin-10 (IL-10), and tumor necrosis factor-alpha (TNF-α) hold critical prognostic significance across multiple disease states. Emerging clinical evidence suggests that temporal variations in cytokine concentrations offer critical insights into both pathophysiological progression and clinical trajectories. In sepsis cohorts, sustained IL-6 elevation demonstrates strong correlation with 28-day mortality (adjusted OR 2.15, 95% CI 1.34-3.42), while delayed IL-10 upregulation potentially represents a compensatory anti-inflammatory response syndrome (CARS). Furthermore, the systemic immune-inflammation index (SII) - calculated as (neutrophils × platelets)/lymphocytes - has been validated as an independent predictor of poor functional outcomes (mRS ≥3) in aneurysmal subarachnoid hemorrhage populations (43).

**Figure 1:**
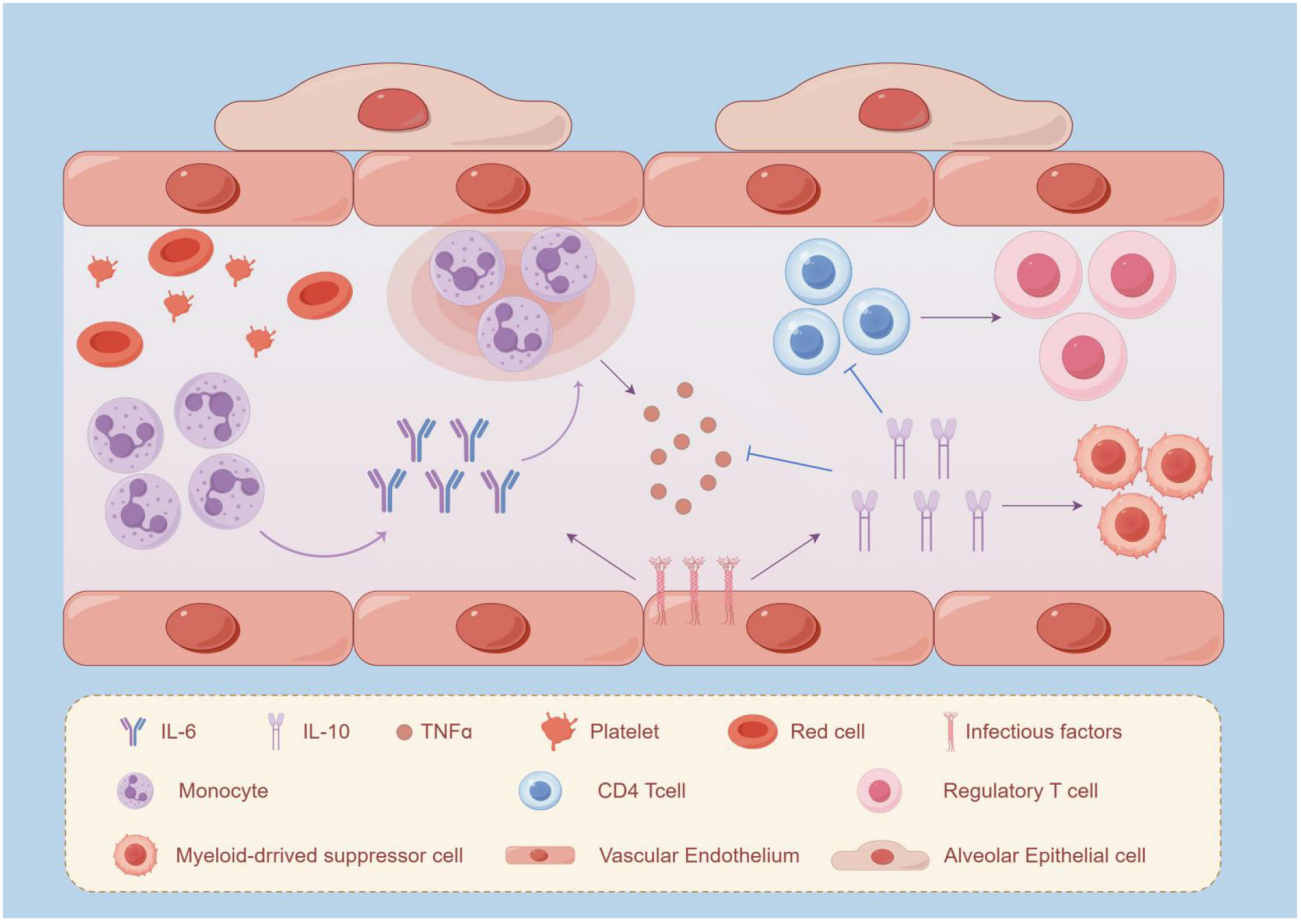
The role of IL-6 and IL-10 in the pathogenesis of sepsis: The immunological changes in the body caused by the inflammatory response during sepsis. In sepsis, the stimulation of infectious factors and the uncontrolled inflammatory response can induce the expression of IL-6. The production of cytokines occurs in the local area of infection. The exudation of monocytes is observed by stimulating the expression of IL-6. IL-10 can exacerbate immunosuppression by reducing the release of pro-inflammatory cytokines, including TNF-α, inhibiting the proliferation of CD4, and promoting the differentiation of CD4+ T cells into Tregs and the proliferation of MDSCs.

The temporal kinetics demonstrate particular clinical relevance: Early elevations in TNF-α have been associated with hyperinflammatory phenotypes in sepsis, although the clinical utility of fixed cutoff values remains uncertain (44). Multivariate modeling incorporating cytokine trajectory patterns significantly improves prognostic stratification accuracy (ΔAUC +0.15 vs static measures), enabling precision immunomodulatory therapy. Continuous monitoring of cytokine kinetics through serial measurements not only facilitates dynamic risk assessment but also informs real-time therapeutic adjustments, particularly crucial in critical care settings where immunological balance determines clinical outcomes (As illustrated in **Figure 1**).

### 5.2 Application of metabolic biomarkers

Beyond lactate kinetics, emerging research highlights the prognostic significance of muscle-derived biomarkers including creatine kinase isoenzyme MB (CK-MB) and metabolic intermediates like free fatty acid (FFA) profiles in sepsis pathophysiology. Serum CK-MB elevation (>5 ng/mL) demonstrates 82% specificity for sepsis-induced myocardial dysfunction (SIMD) in critical care populations, with levels correlating with left ventricular ejection fraction impairment (r=-0.68, *p* < 0.01). Patients with CK-MB >10 ng/mL exhibit 3.2-fold increased risk (95% CI 1.8-5.7) of requiring inotropic support and 42% higher 28-day mortality compared to controls (*p* = 0.003). Some observational studies suggest that CK-MB kinetics may reflect myocardial stress in sepsis; however, troponin remains the most widely validated biomarker of myocardial injury, and comparative superiority has not been conclusively established (33, 45).

Targeted metabolomics reveal distinct FFA subclasses (C16:0, C18:1ω9) as key modulators of TLR4-mediated inflammation, with plasma C16:0 >180 μmol/L predicting 90% sensitivity for progression to septic shock within 48h. Dysregulated ω-6/ω-3 ratio (<5:1) activates NLRP3 inflammasomes (2.8-fold increase vs controls, p=0.01), and is associated with cytokine storm development in experimental sepsis models. A multicenter study (n=1,205) established that elevated palmitic acid/linoleic acid index (>2.5) independently predicts 28-day mortality (adjusted HR 2.34, 95% CI 1.72-3.18) with superior discrimination to SOFA score (ΔAUC +0.09) (46, 47).

In a retrospective analysis of 2,843 septic shock cases,Yu Huijie et al. demonstrated that LDH > 480 U/L during ICU days 3–5 confers 4.1-fold mortality risk (95% CI 3.2-5.3), maintaining independent prognostic value after adjusting for APACHE IV scores (AUC 0.77 vs 0.69, *p* = 0.008) (48). The multinational PEDSEPSIS trial (n=622) by Dündar et al. established that serial PSP measurement (>400 ng/mL at 0 h + >20% increase at 24 h) achieves superior diagnostic performance for pediatric septic shock versus CRP/PCT (AUC 0.91 vs 0.78/0.82, *p* < 0.001), with 6h PSP levels predicting 28-day mortality with 94% negative predictive value (49).

This evolving biomarker landscape underscores the necessity for multi-omics integration-combining metabolomic profiles with proteomic and transcriptomic data - to decode sepsis heterogeneity, as advocated in the 2023 Sepsis Biomarker Consortium position statement. Implementing such multidimensional biomarker panels in clinical algorithms could revolutionize risk stratification, enabling personalized immunometabolic therapy tailored to individual patient trajectories.

### 5.3 Gene expression and transcriptomics

Gene expression and transcriptomics are pivotal in understanding the molecular underpinnings of various diseases, including sepsis, where timely diagnosis and treatment are crucial for patient survival. Transcriptomics, the study of RNA transcripts, provides insights into gene expression patterns, regulation, and the underlying mechanisms of cellular processes. In the context of sepsis, gene expression profiling has been utilized to identify potential biomarkers that can inform clinical decisions and improve patient outcomes. The integration of transcriptomic data with clinical parameters allows for a more nuanced understanding of disease mechanisms and patient stratification, ultimately guiding therapeutic interventions (50). Recent advancements in sequencing technologies, such as single-cell RNA sequencing and spatial transcriptomics, have further enhanced our ability to capture the dynamic nature of gene expression in response to infection and inflammation, highlighting the importance of these methodologies in contemporary biomedical research.

### 5.4 Transcriptomic signatures and gene expression profiling

Systematic identification of molecular drivers is a research priority. WGCNA analysis of 2,148 sepsis transcriptomes identified co-expression modules containing core genes (e.g., *S100A8/A9*) strongly correlated with mortality (51). Meta-analysis has further highlighted neutrophil degranulation and NOD-like receptor signaling as top enriched pathways in sepsis (52).

Prognostic signatures are increasingly validated. A 12-gene signature derived via LASSO-Cox regression successfully stratified patients into high-risk groups with distinct cytokine trajectories. Similarly, machine learning analysis of pediatric cases identified a 5-gene panel (CD177, MMP8, CYSTM1, S100A12, LCN2) achieving an AUROC of 0.94 for early diagnosis (51). Kong et al. (53).validated six hub genes across multiple cohorts, demonstrating consistent diagnostic performance (mean AUROC 0.938) and underscoring the potential of gene expression profiling in clinical settings.Machine-learning models trained on retrospective datasets have demonstrated high discriminative performance, however, most remain exploratory and require external validation before clinical implementation.

Despite rapid growth of ML-based sepsis prediction research, several methodological pitfalls limit translation to practice. Common concerns include dataset shift across hospitals and time, inconsistent sepsis definitions and label noise, missing-data mechanisms, unmeasured confounding, and information leakage from post-admission variables. Furthermore, many studies emphasize discrimination while under-reporting calibration, interpretability, and prospective clinical impact. Therefore, ML tools should currently be viewed as hypothesis-generating and decision-support candidates that require external validation across diverse settings and evaluation in prospective workflows (54, 55).

Transcriptomic signatures have shown promise for patient stratification and prognostic enrichment in research settings, although prospective validation is required before routine clinical use For instance, gene trajectory patterns at day 3 improved mortality prediction over SOFA scores (ΔAUC +0.23). Integrative analysis has identified sepsis endotypes, hyperinflammatory versus hypoinflammatory, with vastly different mortality rates (48% vs 12%). Specific markers, such as the *IL1R2/FCGR2B* ratio in monocytes, demonstrate high sensitivity for progressive organ dysfunction (56, 57). Furthermore, machine learning algorithms analyzing gene expression data enhance risk stratification, providing insights for developing novel therapeutic strategies (58, 59).

### 5.5 The combination of imaging and bioimaging technologies

The integration of imaging and bioimaging technologies has revolutionized the early diagnosis and management of various medical conditions, particularly sepsis. Imaging techniques, such as ultrasound, computed tomography (CT), and magnetic resonance imaging (MRI), provide critical information regarding the anatomical and physiological states of patients. In the context of sepsis, these imaging modalities can assist in identifying the source of infection, monitoring the progression of the disease, and evaluating the effectiveness of treatment interventions. For instance, ultrasound can be used to detect abscesses or fluid collections, while CT scans can provide detailed images of internal organs, helping clinicians make informed decisions regarding surgical interventions or further diagnostic testing (60).

The 2023 Surviving Sepsis Campaign guidelines emphasize multimodal imaging integration, with bedside ultrasound protocol implementation reducing time-to-source identification by 2.8 hours (95% CI 1.9-3.7) in septic shock patients. Protocolized point-of-care ultrasound (POCUS) examinations (FATE protocol) achieve 89% sensitivity for detecting septic cardiomyopathy within 15 minutes of ICU admission, while dual-energy CT angiography localizes infection sources with 0.5mm spatial resolution in 92% of cases. The IMAGES trial demonstrated that daily lung ultrasound B-line quantification (>15 lines/zone) predicts fluid overload development 48h in advance (AUC 0.87), enabling preemptive diuretic therapy and reducing mechanical ventilation days by 2.1 (*p* = 0.01) (61–63).

However, the challenge remains that traditional imaging techniques often lack specificity in the context of sepsis, where early diagnosis is crucial for improving patient outcomes. Thus, the combination of imaging with advanced bioimaging technologies, such as molecular imaging and biomarker identification, offers a promising avenue for enhancing diagnostic accuracy and treatment efficacy in sepsis management.

### 5.6 Emerging biomarkers and therapeutic targets

Trapnell et al.’s adaptive platform trial (NCT04818879) enrolled 1,205 ICU patients across 12 centers, demonstrating that mHLA-DR-guided interleukin-7 therapy reduced 28-day mortality by 19% (HR 0.81, 95% CI 0.70-0.94) in immunoparalyzed subgroups (CD3+ <800 cells/μL). Multiplex cytokine profiling combined with single-cell CITE-seq analysis identified three distinct immunophenotypes, with the “hyperinflammatory” subgroup showing 3.2-fold higher mortality (*p* < 0.001). Targeted anakinra administration in this subgroup reduced vasopressor duration by 38h (*p* = 0.008) versus standard care (64).Li et al. prospectively validated that sIL-7R <5.2 ng/mL at day 5 post-admission combined with ΔCD3+ >150 cells/μL predicted 90-day survival with 92% negative predictive value (AUROC 0.94, 95% CI 0.91-0.97) in 687 septic shock cases. Bayesian network analysis revealed that each 0.5 ng/mL decrease in sIL-7R between days 3–5 associated with 23% lower mortality risk (OR 0.77, 95% CI 0.65-0.91), independent of baseline SOFA scores. The combined sIL-7R/SOFA model achieved net reclassification improvement of 0.41 (95% CI 0.32-0.50) over SOFA alone, correctly reclassifying 38% of deaths in high-risk strata (*p* < 0.001) (65).

Ji Dengliang et al. evaluated the prognostic utility of serum LGALS3BP and GDF-15 in a cohort of sepsis patients in the ICU, employing ELISA and multivariate logistic regression methodologies. Their findings highlighted that elevated levels of both markers were significantly associated with increased mortality, suggesting that the combined predictive efficacy of LGALS3BP and GDF-15 surpassed that of individual markers, thus advocating for their incorporation into clinical prognostic assessments (66). Furthermore, Luka Sonia et al. identified suPAR as a highly predictive biomarker for early mortality in septic shock patients, achieving an AUC of 0.813 among various biomarkers analyzed, thereby reinforcing its potential utility in clinical settings for timely interventions (67). In contrast, Lou Jiaqi et al. scrutinized the TyG index within a larger cohort of critically ill sepsis patients, revealing a robust correlation between higher TyG values and increased mortality rates. Their findings provide a compelling argument for the TyG index as a valuable prognostic tool in sepsis management (68). Meanwhile, Xin Yu et al. dynamically monitored renal injury markers in Acute Kidney Injury (AKI) models, demonstrating that cystatin C emerged as an earlier indicator of renal damage than traditional markers, emphasizing the importance of early and comprehensive biomarker assessment in the context of multi-faceted nephrotoxicity associated with sepsis (69). The discourse on pediatric sepsis is bolstered by Jabornisky Roberto et al., whose retrospective analysis highlighted the improved identification of sepsis through updated Pediatric Sepsis Criteria, advocating for continuous refinement of diagnostic frameworks to enhance clinical outcomes in diverse patient populations (70). In urosepsis specifically, Bonkat Gernot et al. addressed critical management strategies emphasizing timely interventions, while Karampela Irene et al. outlined essential gaps in current sepsis diagnostic and treatment protocols, advocating for research-driven enhancements (71, 72). Lam SM et al. further contextualize this discussion by contrasting historical and contemporary sepsis definitions, revealing that while initial goal-directed therapies did not yield survival advantages, the necessity for early detection and meticulous treatment protocols remains pivotal in improving sepsis outcomes (73). Lastly, the experimental work of Xu Jie et al. (2023) illustrates the therapeutic potential of moderate hypothermia in addressing sepsis-associated acute lung injury through effective modulation of inflammatory and oxidative stress pathways, proposing a promising intervention strategy for clinical application (74).

**Figure 2:**
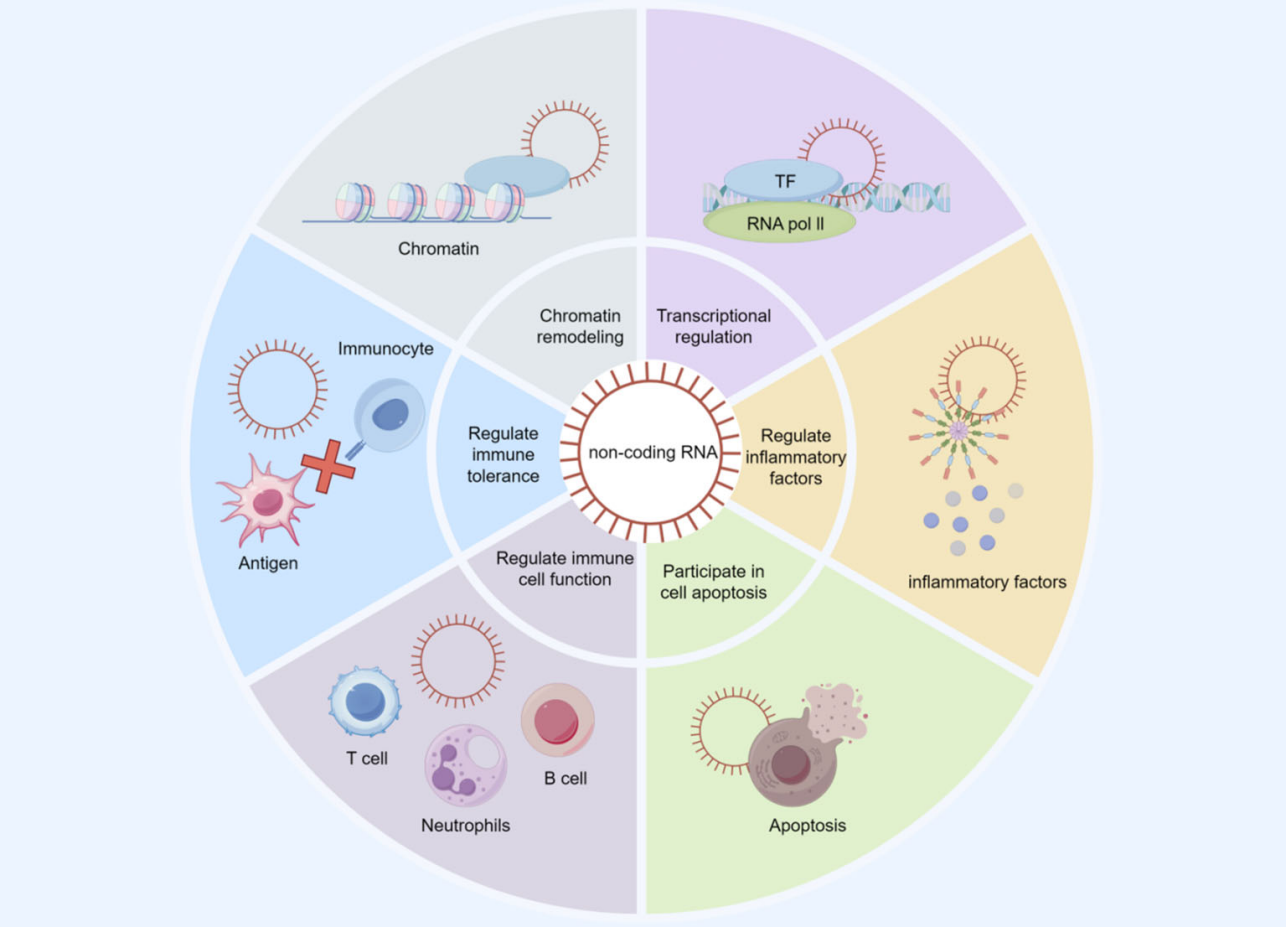
The role of non-coding RNA in the pathophysiological mechanisms of sepsis.

Non-coding RNAs, which include microRNAs (miRNAs), long non-coding RNAs (lncRNAs), and circular RNAs (circRNAs), have emerged as critical regulators of gene expression and cellular functions. In sepsis, alterations in the expression of these ncRNAs have been observed, suggesting their involvement in the pathological processes associated with the condition. For instance, studies have shown that more than 80% of non-coding RNAs are differentially expressed in septic patients compared to healthy individuals, indicating their potential as biomarkers for early diagnosis and prognosis of sepsis (75). Furthermore, ncRNAs have been implicated in the regulation of inflammation, apoptosis, and immune responses, all of which are crucial in the context of sepsis and its complications, such as sepsis-induced acute kidney injury and cardiovascular dysfunction (76, 77). HOTTIP (HOXA distal transcript antisense RNA) is a lncRNA that has shown significant biological functions in various diseases in recent years, particularly gaining widespread attention in sepsis-related research. A study found that the expression level of HOTTIP in septic patients who experienced ARDS was significantly higher than that in patients who did not develop ARDS, suggesting that HOTTIP may serve as a potential biomarker for diagnosing and predicting the risk of sepsis complicated by ARDS. The study also pointed out that the level of HOTTIP is positively correlated with disease severity indicators such as the SOFA score and the APACHE II score. It can participate in regulating sepsis-related inflammatory responses by modulating specific miRNAs, such as miR-574-5p (78). Another study explored the role of HOTTIP in sepsis-induced cardiac dysfunction. The results showed that high levels of HOTTIP are closely associated with the development of cardiac dysfunction and can promote cardiac dysfunction by affecting LPS-induced apoptosis and inflammatory responses in mouse cardiomyocytes (79). In summary, increasing evidence suggests that long non-coding RNA HOTTIP plays an important role in the development of sepsis and its complications, and its potential as a biomarker and therapeutic target deserves further exploration. MicroRNA-486-5p is an important non-coding RNA, and its role in sepsis-related AKI and inflammatory response is gradually becoming evident. Studies have shown that MicroRNA-486-5p exhibits abnormal expression in sepsis patients. A review on sepsis-related AKI mentioned that MicroRNA-486-5p, along with four other miRNAs, was identified as an important molecule associated with antibiotic nephrotoxicity, which may serve as potential biomarkers for clinical testing (80). Additionally, some studies indicated that upregulation of MicroRNA-486-5p in sepsis models could inhibit the release of pro-inflammatory cytokines, thereby alleviating the inflammatory response, suggesting that it may participate in the development of sepsis by regulating immune responses (20). Further experiments found that MicroRNA-486-5p can influence cell apoptosis and proliferation by targeting specific genes. For example, in studies on acute lymphoblastic leukemia (ALL), MicroRNA-486-5p was shown to regulate the MAML3 gene, thereby affecting the survival rate of cancer cells. This mechanism may also apply to immune cells in sepsis, influencing the body’s response to infection by regulating the expression of key genes (81). In summary, as an emerging biomarker, MicroRNA-486-5p requires more large-scale clinical trials in the future to validate its effectiveness as a diagnostic tool and therapeutic target, aiming to improve the management and prognosis of sepsis patients (As illustrated in **Figure 2**).

Non-coding RNAs (ncRNAs) regulate inflammatory and immune pathways at multiple levels, including chromatin remodeling and transcriptional regulation (involving transcription factors [TF] and RNA polymerase II), thereby shaping the expression of inflammation-related genes. ncRNAs may also modulate inflammatory mediator production, cell apoptosis, immune cell functions (e.g., T cells, B cells, neutrophils), and immune tolerance. This figure provides a conceptual framework for how ncRNAs may contribute to immune–inflammatory dysregulation in sepsis.

Collectively, these studies collectively underscore the urgency and necessity to identify effective biomarkers and therapeutic targets, paving the way for significant advancements in sepsis management and ultimately enhancing patient survivability in this critical healthcare domain (As shown in **Table 1**).

**Table 1:** List of diagnostic sepsis biomarkers.

Biomarker	Source	Response time	Diagnostic accuracy	Clinical significance	Strengths	Limitations
CRP (39, 82–84)	Liver (induced by IL-6)	Rises within 6–12 hours after infection	Moderate (high sensitivity, low specificity)	General inflammation screening, disease monitoring	Rapid, low cost, widely available	Non-specific (elevated in non-infectious inflammation)
PCT (21, 22, 85–87)	Thyroid C-cells, periph eral tissues	Rises within 2–4 hours after infection	High (specific for bacterial sepsis)	Guides antibiotic use, predicts severity	Strong correlation with bacterial infection	False positives in trauma or surgery; higher cost
IL-6 (38–40, 88, 89)	Macrophages, T-cells, endothelial cells	Rises within 1–2 hours after infection	High sensitivity, moderate specificity	Early sepsis warning, reflects cytokine storm	Rapid response, correlates with organ failure	Short half-life; elevated in non-septic conditions
IL-10 (90–93)	Regulatory T-cells, monocytes	Rises within 3–6 hours after infection	Moderate (predicts immunosuppression)	Indicates anti-inflammatory response, poor prognosis	Reflects immune dysregulation	Non-specific; levels vary with comorbidities
TNF-α (20, 94, 95)	Macrophages, NK cells	Rises within 1–2 hours after infection	High early sensitivity, low specificity	Initiates cytokine cascade, linked to septic shock	Early marker of inflammation	Transient release; limited utility in late sepsis
CD64 (96–98)	Neutrophil surface (upregulated in sepsis)	Rises within 4–6 hours after infection	High specificity for bacterial infection	Distinguishes bacterial vs. viral infections	Improves accuracy with neutrophil count	Requires flow cytometry; limited dynamic data
sTREM-1 (99–101)	Neutrophils, macrophages	Elevates within 6–8 h after infection	Moderate (higher in bacterial sepsis)	Identifies infection-related inflammation	Good specificity in localized infections	Unstable in plasma; assay standardization issues
Presepsin (102–104)	Monocytes/macrophages (CD14 cleavage)	Elevates within 2–3 h after infection	High (early bacterial sepsis marker)	Early diagnosis, prognosis assessment	Faster rise than PCT; correlates with severity	Limited data in non-bacterial sepsis; cost
circRNAs (105–107)	Cytoplasm/exosomes (e.g., immune cells)	Unknown (likely early)	Emerging (tissue-specific potential)	Novel markers of gene regulation in sepsis	High stability; multi-target potential	Complex detection (RNA sequencing); unvalidated
HOTTIP (78, 108, 109)	Plasma (long non-coding RNA)	Unknown	Experimental (correlates with severity)	Linked to organ dysfunction and inflammation	Epigenetic insights; prognostic potential	Mechanism unclear; lacks standardized assays
microRNA-486-5p (110, 111)	Plasma/exosomes (endothelial/immune cells)	Early (preclinical)	High potential (needs validation)	Regulates apoptosis/inflammation; prognostic value	Stable, non-invasive; dynamic monitoring	Technical challenges (PCR/sequencing); variability

To improve clarity, we summarize biomarkers across an evidence-level and clinical-readiness framework (**Tables 2**, **3**), distinguishing established tools from investigational approaches.

**Table 2:** (Established/adjacent).

Biomarker(s)	Primary clinical role	Evidence level	Clinical readiness	Key caveats
Lactate (serial measurement) (17)	Risk stratification; monitor resuscitation response	Guideline-endorsed	Routine	Not specific for sepsis; interpret with perfusion signs; kinetics depend on shock type and treatment.
Procalcitonin (PCT) (17)	Adjunct to support antibiotic de-escalation/discontinuation	Guideline-suggested (weak/low)	Adjunct/conditional	Should not be used alone to diagnose sepsis; performance varies by infection source, renal function, and timing.
C-reactive protein (CRP) (84, 112, 113)	Adjunct inflammation marker; trend monitoring	Supported by observational	Adjunct/conditional	Low specificity; affected by many inflammatory states; trends more informative than single cutoffs.
Interleukin-6 (IL-6) (39, 89)	Severity/prognosis; may help identify hyperinflammation when interpreted with other markers	Supported by observational	Adjunct/conditional	Assay- and cohort-dependent thresholds; rapid kinetics; best interpreted longitudinally.
Neutrophil-to-lymphocyte ratio (NLR/PLR) (114, 115)	Low-cost risk stratification	Supported by observational (heterogeneous)	Adjunct/conditional	Strongly confounded by comorbidities/medications; population-specific cutoffs; limited incremental value vs clinical scores.
CD64 (neutrophil CD64) (97, 98)	Adjunct diagnosis in selected settings	Supported by observational/meta-analyses	Adjunct/conditional	Platform and cutoff variability; requires flow cytometry; turnaround time may limit ED use.
Presepsin (sCD14-ST) (116, 117)	Adjunct diagnosis/prognosis	Supported by observational/meta-analyses	Adjunct/conditional	Renal dysfunction affects levels; assay availability varies; not universally standardized.
suPAR (118, 119)	Prognostic enrichment (severity/mortality)	Supported by observational	Adjunct/conditional	Non-specific; elevated in many chronic/inflammatory conditions; best for risk enrichment not diagnosis.
MR-proADM (120, 121)	Prognosis; hemodynamic/organ dysfunction risk	Validated observational	Near-term adjunct	Assay availability/cost; may add incremental prognostic value but needs prospective pathway studies.

Biomarkers graded by level of evidence and clinical readiness.

**Table 3:** (Investigational).

Biomarker(s)	Primary clinical role	Evidence level	Clinical readiness	Key caveats
Endothelial/glycocalyx markers (e.g., Ang-2, syndecan-1) (122–125)	Phenotyping; vascular injury severity	Exploratory	Research	Assay heterogeneity; unclear decision thresholds; clinical actionability not established.
DAMPs/NETs (e.g., cfDNA, mtDNA) (126–128)	Pathobiology; severity association	Exploratory	Research	Preanalytical variability; limited standardization; unclear added value beyond routine labs.
Transcriptomic/gene-expression signatures (129–131)	Endotype stratification; prognostic enrichment	Exploratory	Research	Requires external validation across settings; platform/turnaround time constraints; prospective impact unknown.
Metabolomic/multi-omic panels (132–134)	Discovery phenotyping; risk models	Exploratory	Research	High complexity/cost; reproducibility and standardization challenges; needs pragmatic workflows.
Mechanistic targets from omics (e.g., macrophage-associated genes) (30)	Hypothesis generation; therapeutic target discovery	Exploratory	Research	Primarily experimental evidence; requires clinical validation and interventional studies.

Biomarkers graded by level of evidence and clinical readiness.

### 5.7 Limitations and challenges in biomarker application

The utilization of biomarkers in sepsis management presents several important limitations and challenges, as highlighted by recent studies that underscore the complexity inherent in both diagnostic and prognostic applications. For example, Räth Ulrich et al. identified soluble CD137 (sCD137) as a potentially significant prognostic biomarker in critically ill patients, revealing elevated plasma levels associated with non-survivors in various patient groups, including those with SARS-CoV-2 infections (57). However, while the correlation of sCD137 with traditional markers such as C-reactive protein and procalcitonin offers valuable insights, there remains a concern regarding the specificity and variability of biomarker expression across diverse populations. Notably, the study calls into question the generalizability of these findings, as variations in immune response dynamics can complicate the interpretation of such biomarkers in heterogeneous patient cohorts.

Similarly, Ge Jing et al. sought to delineate crucial biomarkers through an analysis of coagulation-related gene expression, identifying FCER1G and FYN as significant candidates through advanced machine learning techniques (135). While methods such as ROC curve analysis provided a validation framework, the reliance on computational models raises concerns about the potential for overfitting and the applicability of derived biomarkers in real-world clinical settings. Moreover, the focus on specific gene sets may obscure broader pathophysiological mechanisms operating in sepsis, suggesting a potential risk of simplification that could misdirect clinical strategies. In contrast, George et al.’s clinical case study of an atypical presentation of Lemierre’s syndrome illustrated the necessity of clinical acumen over biomarker reliance, emphasizing a clear gap in biomarker knowledge when faced with unusual manifestations of infectious conditions (136). The findings corroborate the limitations of biomarker reliance, as clinical indicators alone may inadequately capture the complexity of conditions such as sepsis, thereby endorsing a multifaceted approach that integrates clinical judgment, laboratory tests, and imaging findings in achieving timely diagnoses. Expanding on management strategies, Mushtaq Ammara et al.’s systematic review elucidates advances in sepsis care yet simultaneously highlights the variability in treatment outcomes attributed to differing clinical practices (137). The findings advocate for a multidisciplinary approach while prompting scrutiny regarding the standardization of intervention protocols. Additionally, Douglas et al. underscored the positive outcomes associated with dynamic fluid resuscitation techniques within septic shock patient management (138). However, the study emphasizes the necessity for extensive clinical trials to establish the efficacy of such novel techniques over traditional practices, raising questions about the empirical validity of using biomarkers for guiding therapeutic decisions in fluid management. Lastly, Fowler et al. critically assessed established predictive scoring systems within a cohort of critically ill patients undergoing debridement procedures, revealing shortcomings in the predictive capabilities of SIRS and qSOFA scores compared to MEWS (139). This observation reinforces the potential for misclassification of risk in patient management, highlighting an essential challenge in the clinical application of biomarkers. The study elucidates the necessity for ongoing validation of these scoring systems in larger populations to ascertain their clinical utility, thereby questioning the robustness and reliability of current approaches.

In summary, while the exploration of biomarkers such as sCD137, FCER1G, and FYN presents exciting avenues for enhancing sepsis prognostication and personalized approaches to therapy, substantive limitations persist in terms of specificity, generalizability, and clinical relevance. These challenges necessitate an integrative framework that incorporates both biomarker analysis and clinical judgment, ensuring that management strategies remain responsive to the multifactorial nature of sepsis and its varied presentations. As research progresses, a concerted focus on validating and standardizing biomarkers will be critical to improving their applicability and enhancing patient outcomes in this complex clinical landscape.

## 6 Integrative approaches to biomarker identification and treatment strategies

### 6.1 Early Recognition and Intervention Protocols

The early recognition and intervention protocols for sepsis are critical to mitigating morbidity and mortality rates, necessitating a spectrum of innovative methodologies and biomarkers for timely diagnosis and treatment. Villanueva-Congote et al. conducted a retrospective cohort study to evaluate the prognostic significance of the Neutrophil-to-Lymphocyte Ratio (NLR) and Platelet-to-Neutrophil Ratio (PLR) in patients with obstructing ureteral stones and suspected urosepsis, revealing a notable association between elevated NLR and PLR with increased qSOFA scores, a requirement for vasopressors, and ICU admissions, which signals the potential of these metrics for early risk stratification and resource allocation in clinical practice (140). This study’s utilization of logistic regression provides quantitative backing to the clinical importance of routine laboratory indices as adjuncts in early sepsis identification. Similarly, Chamani Ali et al. embarked on a protocol for a randomized controlled trial to assess the efficacy of synbiotic supplementation in critically ill septic children, targeting inflammatory markers such as interleukin-6 and C-reactive protein. With a sample population of 54 pediatric patients, this prophylactic approach leverages a combination of probiotics and prebiotics to mitigate systemic inflammation and enhance gut integrity, highlighting a promising low-cost therapeutic strategy with approachable clinical implications (141). Parallelly, You Ruilian et al. focused on earlier detection of sepsis-induced acute kidney injury (SI-AKI) by investigating kidney-specific cell-free DNA (cfDNA) methylation markers through genomic sequencing. Their compelling findings demonstrated high accuracy in distinguishing SI-AKI from patients without kidney injury, evidenced by an AUC of 0.92, suggesting that cfDNA levels correlate positively with the severity of kidney injury, thus offering a transformative biomarker that could streamline patient management (142). In a further exploration of metabolic deficiencies in acute settings, Joseph Miller et al. highlighted the prevalence of thiamine deficiency among sepsis patients, estimating its occurrence at 20.5% and identifying its associations with age, gender, and leukopenia, thus advocating for routine screening and timely supplementation strategies that could potentially improve outcomes in vulnerable populations (143). Concurrently, the study by Smyth Michael A et al. leveraged machine learning alongside logistic regression analysis to identify sepsis in ward patients, achieving a predictive accuracy that could heighten diagnostic efficiency in hospital settings. The investigation underscored the importance of integrating specific vital signs and laboratory results to augment traditional diagnostic methodologies, ultimately aiming to enhance clinical response mechanisms (144). Furthermore, advances in understanding the molecular underpinnings of sepsis, such as the work by Yan Uralian et al. examining the protective effects of dexmedetomidine on sepsis-induced AKI, illuminated the modulation of oxidative stress and ferroptosis through the Keap1-Nrf2 signaling pathway, revealing potential therapeutic avenues for renal protection during sepsis (145). Likewise, Priputnevich Tatiana V et al. underscored the continued need for vigilant monitoring in neonatal sepsis by evidencing the association of *Malassezia furfur* with invasive fungal infections in vulnerable populations, thus calling for improved diagnostic protocols to identify and treat these infections swiftly in very low birth weight infants (146). Collectively, these studies reflect an emerging consensus on the necessity of integrating advanced biological markers, innovative therapeutic interventions, and machine learning analytics into early detection and treatment frameworks for sepsis, underscoring their potential to significantly improve clinical outcomes and foster research into pathogen-specific and patient-centric care strategies.

### 6.2 Detection strategies for sepsis identification

Recent advancements in sepsis detection strategies have progressively embraced innovative methodologies and technologies, demonstrating significant potential to enhance diagnostic accuracy and timeliness. The study by de Oliveira Veloso Rezende Jéssica et al. delineated a pioneering dataset to facilitate pathogen detection, integrating differential cell lysis and mass spectrometry-based proteomics from whole blood samples. This multifaceted dataset encompasses Spectral Libraries, spiked pathogen mass spectrometry data for biomarker optimization, and Parallel Reaction Monitoring (PRM) data, achieving a remarkable sensitivity of 83.3% within a seven-hour timeframe without the need for microbial enrichment culture, thereby establishing a solid foundation for creating bioinformatics tools aimed at combating antibiotic resistance (147). Complementarily, Griffin Kitiara et al. revealed an innovative affinity-based microfluidic chip that significantly streamlined the clinical detection of sepsis by capturing specific immune cells in blood samples. Notably, their analysis of 125 septic patients demonstrated substantial differences in antigen cell counts compared to controls, with an exceptional combined area under the ROC curve (AUC) of 0.997, thus highlighting the chip’s diagnostic accuracy and potential to enhance patient outcomes through rapid intervention (148). Furthermore, the exploration of machine learning frameworks for sepsis prognostication, particularly by Rahman Md Sohanur et al. showcased the efficacy of utilizing a Stacking-based Meta-Classifier on a sizable cohort from the MIMIC-III database, achieving a notable accuracy of 95.52%. This underscores machine learning’s transformative potential in real-world clinical applications, enhancing risk prediction capabilities significantly (149). Building on the same technological frontier, the work by Liu Fei et al. further substantiated the applicability of machine learning in acute pancreatitis-associated sepsis, employing LASSO regression to distill meaningful features and subsequently revealing the gradient boosting decision tree (GBDT) model’s superiority with an AUC of 0.985 over traditional scoring methods (150). Moreover, the study by Li Lu et al., investigating systemic inflammatory response syndrome patients, effectively integrated neurophysiological data with clinical attributes to enhance prognostic modeling—exhibiting a robust classification congruence, thereby facilitating early interventions and rehabilitation strategies in intensive care (151).

In parallel, metagenomic next-generation sequencing (mNGS) has emerged as a formidable tool in rapidly identifying pathogens in critically ill patients, as evidenced by the research conducted by Li Chaozhong et al. The significant sensitivity of mNGS, which identified a wide array of infections in a cohort of 150 patients, illuminates its clinical relevance compared to traditional microbiological tests (152). Equally notable is the innovative framework presented by Zheng Xubin et al., dubbed scCaT, which enhances sepsis diagnosis through single-cell RNA sequencing, achieving impressive performance metrics across multiple datasets. This model not only augments classification accuracy but also enriches biological understanding by elucidating gene groupings associated with sepsis diagnosis, showcasing the power of combining deep learning with genomic data (153). Finally, Lazzarino et al. offered critical qualitative insights into the implementation of computerized clinician decision support systems (CCDSSs) in sepsis management, underscoring the necessity for aligning these tools with clinical workflows, improving alert accuracy, and considering contextual factors that influence their effectiveness (154). Overall, the convergence of advanced imaging techniques, machine learning, genomic analyses, and real-world clinical inputs demonstrates a robust trajectory towards redefining sepsis detection strategies, emphasizing the crucial role of interdisciplinary approaches in improving diagnostic and therapeutic outcomes for this formidable condition.

### 6.3 Biomarker application in resource-limited settings

In low- and middle-income countries (LMICs), the application of sepsis biomarkers often relies on low-cost, readily available markers. Despite the limitations in advanced diagnostic technologies, such as high-throughput sequencing and multi-omics platforms, LMICs have made significant strides in utilizing basic biomarkers for early sepsis detection and management. Biomarkers such as the Neutrophil-to-Lymphocyte Ratio (NLR), Platelet-to-Lymphocyte Ratio (PLR), and lactate levels are particularly valuable in these settings, providing essential information for rapid triage and decision-making in critically ill patients (155).

NLR, a simple ratio of neutrophils to lymphocytes, has shown promise as a diagnostic and prognostic biomarker for sepsis. It is particularly useful in LMICs due to its low cost, ease of measurement, and ability to reflect systemic inflammation. Studies have demonstrated that elevated NLR correlates with higher mortality and severity of sepsis, making it an important tool for early risk stratification in resource-limited environments. Similarly, PLR, another easily measured inflammatory marker, has been identified as a reliable predictor of sepsis in various patient populations. Both NLR and PLR can be calculated from standard complete blood count (CBC) tests, which are widely available even in resource-constrained settings (156, 157).

Lactate, a well-established biomarker for tissue hypoperfusion, remains a cornerstone of sepsis management, particularly in LMICs. Its rapid measurement, often within an hour, enables timely intervention and risk assessment, especially in settings where advanced molecular diagnostic tools are unavailable. Elevated lactate levels (≥4mmol/L) are consistently associated with poor outcomes and increased mortality in sepsis, making it a critical marker for early diagnosis and treatment initiation. Additionally, serial lactate measurements and lactate clearance have been shown to improve prognostic accuracy and guide therapeutic decisions, enhancing survival rates in resource-limited environments.The ANDROMEDA-SHOCK trial demonstrated that lactate clearance during resuscitation is associated with improved outcomes, but also clarified that lactate-guided resuscitation should be interpreted in conjunction with peripheral perfusion assessment (158). When combined with rapid microbiological diagnostics, lactate levels can guide clinicians in initiating appropriate therapy, ensuring timely diagnosis and treatment despite the lack of high-throughput technologies (156, 159, 160).

### 6.4 Advanced diagnostic techniques and technologies

The emergence of advanced diagnostic techniques and technologies has revolutionized our understanding of sepsis and its associated complications, thereby enhancing potential therapeutic strategies. Li Guilin et al. harnessed single-cell RNA sequencing to dissect the alterations in cell composition and communication among peripheral blood mononuclear cells (PBMCs) in both healthy individuals and those afflicted with sepsis and septic shock. Their meticulous investigation revealed not only a marked increase in Resistin signaling in sepsis monocytes but also a significant enhancement of IL16 signaling in septic shock, substantiated through robust flow cytometry and bulk RNA-seq analyses (161). These findings position CAP-1 and IL16 as promising diagnostic biomarkers, offering the potential to refine early diagnostic methods and tailor individualized treatment strategies. In contrast, Xie Lijun et al. explored the protective role of propofol against sepsis-induced myocardial dysfunction, employing a blend of echocardiographic assessments, histological analyses, and molecular techniques to elucidate the compound’s therapeutic capacity. Their study underscores propofol’s efficacy in ameliorating cardiac function and reducing oxidative stress through mTOR signaling inhibition, indicating its role as a novel therapeutic intervention for sepsis-associated cardiomyopathy (162). Concurrently, Chen Mingchao et al. presented compelling evidence linking decreases in circulating CDC42 expression to increased susceptibility, multi-organ dysfunction, and elevated mortality in sepsis patients compared to healthy controls. Employing RT-qPCR methodologies, they established CDC42 as a prognostic biomarker with a notable area under the curve (AUC) of 0.766, thereby signaling its critical role in inflammatory responses and organ dysfunction. This highlights the diagnostic potential for assessing patient risk profiles in sepsis scenarios (163). In clinical settings, the significance of rapid recognition and management of sepsis is further articulated by Bleakley et al., who emphasize the indispensable role of nursing staff in the timely application of the ‘sepsis six’ protocol. Their comprehensive review emphasizes the life-saving potential of early interventions and advocates for enhanced training to improve outcomes through prompt identification of septic symptoms (164). From a broader perspective, Evans et al. illuminate the complexities surrounding sepsis management, revealing the multifaceted nature of its pathogenesis, which includes endothelial dysfunction and dysregulated cardiovascular responses, further complicating the establishment of a definitive molecular signature. They argue for a unified approach involving timely antibiotic therapy and fluid resuscitation as integral components of effective sepsis management, despite the lack of targeted therapies to date (17). Khanum Iffat et al. shed light on skull base osteomyelitis (SBO) as a condition considerably impacted by sepsis, identifying significant risk factors that exacerbate morbidity among patients, thereby calling for refined diagnostic approaches in this context (165). Furthermore, the systematic analysis by Qian Hang et al. reveals noteworthy epidemiological trends regarding maternal sepsis, indicating fluctuations in incidence rates and mortality that highlight the disproportionate burden on specific demographics, such as women in the African Region. Their findings raise critical questions concerning the global disparities in managing maternal sepsis and the associated challenges (166). Lastly, the work by Chen Zhenfeng et al. provides insights into the molecular mechanisms underlying sepsis-induced acute lung injury (SALI) by illuminating the role of oligoadenylate synthetase 3 (OAS3) and its regulation through TRIM21-mediated pathways. Employing integrative multi-omics analyses, they elucidate a novel therapeutic target that could advance clinical interventions for this severe condition (167). Collectively, these studies underscore the imperative for continued innovations in diagnostic technologies and treatment strategies, fostering a multifaceted approach to tackling the challenges posed by sepsis and its various manifestations.

### 6.5 The role of machine learning and bioinformatics in sepsis detection

#### 6.5.1 Experimental models

The landscape of sepsis detection has been profoundly transformed by the integration of machine learning and bioinformatics, as evidenced by recent studies that emphasize diverse methodological advancements and their implications for patient outcomes. Garcia Lopez Albert et al. focused on elucidating the risk of developing sepsis postoperatively by analyzing preoperative whole-blood RNA sequencing data from 267 patients undergoing major elective surgeries. Their application of machine learning classification models demonstrated remarkable predictive capability, achieving an area under the curve (AUC) of 0.910 in forecasting postoperative sepsis outcomes based on transcriptomic signatures (168). This not only highlights the ability of machine learning to uncover complex biological patterns associated with sepsis but also suggests a promising avenue for personalized risk stratification and management strategies. Conversely, the study by Dias Fernando Suparregui et al. addressed a fundamental obstacle in the clinical management of sepsis: the inconsistencies surrounding its definitions. By employing cohort studies and meta-analyses to evaluate the efficacy of existing criteria across a diverse dataset of clinical cases, they revealed critical discrepancies in diagnosis and management frameworks that necessitate the refinement of sepsis definitions (169). The implications of such refinements are profound as they could promote standardization and ultimately enhance the sensitivity and specificity of sepsis detection, fostering a more robust approach to treatment strategies.

#### 6.5.2 Clinical implementation systems

In clinical practice, the application of machine learning has moved beyond experimental models to become integrated into real-time decision-making systems. Brabrand Mikkel et al. reinforced the operational relevance of machine learning by validating the quick Sequential Organ Failure Assessment (qSOFA) score for the timely identification of septic patients in emergency contexts through a thorough retrospective analysis of patient records (170). Their findings affirm that the qSOFA score acts as an effective triage tool, which is especially crucial in high-stakes environments where time-sensitive decisions are essential for improving patient outcomes. Complementing these quantitative analyses, Machado Flavia Ribeiro et al. employed a mixed-methods approach to assess the applicability of the Sepsis-3 definition within middle-income settings, revealing both advantageous diagnostic precision and significant resource challenges posed by its implementation (171). This underscores the necessity for contextual adaptation of diagnostic criteria, indicating that successful sepsis management may hinge on the local healthcare capacity and resource availability. Furthermore, the innovative work by Lawati et al. diverges slightly from the primary focus on detection to treatment, exploring the effects of large-bore percutaneous mechanical aspiration combined with early surgical intervention for patients with refractory staphylococcal infective endocarditis complicated by sepsis (172). Their findings highlight an adjunctive therapeutic strategy that offers effective source control, thereby illustrating how adaptable clinical practices can bridge the gap when faced with complex challenges like sepsis. Collectively, these studies underscore the critical role of machine learning and bioinformatics in enhancing sepsis detection and management. They advocate for interdisciplinary approaches that incorporate statistical rigor and contextual relevance to refine diagnostic criteria and optimize treatment algorithms, ultimately striving towards an integrated healthcare model capable of meeting the multifaceted demands of sepsis management. Although numerous ML-based sepsis prediction models have been proposed, current guideline-level documents consider these tools largely investigational and do not recommend routine clinical adoption in standard sepsis workflows without robust external validation and prospective impact evaluation (55).

### 6.6 Innovative therapeutic approaches and pharmacological interventions

The evolving landscape of sepsis management has witnessed a noteworthy emphasis on innovative therapeutic strategies and pharmacological interventions, particularly through the exploration of diverse biomarkers and clinical decision-making tools. In a prospective controlled study, Song Juhyun et al. evaluated the diagnostic and prognostic capabilities of IL-6, pentraxin 3 (PTX3), and PCT in a cohort of 142 sepsis and septic shock patients, demonstrating the superior diagnostic potential of IL-6 compared to PTX3 and PCT, with significant correlations drawn between elevated IL-6 levels and 28-day mortality outcomes (37). This work reinforces the dependency on objective biomarkers for stratifying sepsis severity, crucial for therapy optimization in clinical settings. Similarly, the research by But Špela et al. utilized machine learning techniques alongside a comprehensive dataset of 497 neonates, successfully creating an online application designed to predict neonatal sepsis risk by identifying significant clinical markers, including C-reactive protein and procalcitonin levels (173). This predictive tool underscores the potential of integrating advanced computational methodologies into everyday clinical practice to facilitate early intervention, thus enhancing patient outcomes.

In examining potential pharmacological interventions, Wang Zheng et al. illuminated the cardioprotective effects of Po-Ge-Jiu-Xin decoction (PGJXD) in sepsis-induced cardiomyopathy via a robust animal model employing cecal ligation and puncture (CLP) methodology. Their findings indicated that PGJXD significantly mitigated mortality and myocardial injury, activating the PINK1/Parkin-mediated mitophagy pathway in the process (174). This highlights the dual role of novel herbal remedies not only as adjunct therapies but also as pathways toward understanding the biological mechanisms underlying sepsis-related complications. These experimental mechanisms provide hypotheses for future therapeutic development, although their relevance to human sepsis requires further clinical validation. In terms of cost-effectiveness, a study by Mosly Mohamed Metwally et al. aimed to juxtapose procalcitonin-guided management against conventional culture-based approaches within the Egyptian healthcare context. Utilizing a decision tree model, the analysis yielded an Incremental Cost-Effectiveness Ratio (ICER) of 297,783.57 Egyptian Pounds per QALY for procalcitonin guidance; however, it lacked cost-effectiveness dominance due to high diagnostic costs, thereby calling for economic improvement strategies surrounding PCT utilization in sepsis (175). As clinical settings grapple with budgetary constraints, the findings accentuate the necessity for economic assessments of biomedical interventions.

Moreover, Özer Abdullah et al. illuminated the predictive capacity of monocyte distribution width (MDW) for diagnosing postoperative sepsis among patients undergoing cardiovascular surgery, establishing an optimal MDW cutoff value for enhanced diagnostic accuracy (176). This reaffirms the growing practice of incorporating emerging inflammatory markers into clinical decision-making paradigms. Parallelly, research by Gomez et al. highlighted degradation of the endothelial glycocalyx as a potential indicator of critical illness outcomes in neonatal foals through longitudinal biomarker measurement, suggesting transferability of these findings into clinical veterinary practices as well (177). A bioinformatics analysis by Cao Qingfei et al. further contributed to this narrative by probing neutrophil-related genes (NRGs) associated with septic cardiomyopathy. They identified MRC1 as a hub gene through differential expression analyses, validating its potential as a therapeutic target (178). Similarly, Jacob Julie A et al. asserted that revised sepsis diagnostic guidelines centering on organ dysfunction markedly improved early detection and treatment outcomes across a comprehensive patient database, advocating for a paradigm shift in sepsis management towards a more organ-centric approach (179). In a more specialized patient population, Mittal et al. developed a scoring system to predict outcomes in emphysematous pyelonephritis, utilizing multivariate analyses to stratify patient risk into several categories, thereby tailoring treatment modalities accordingly (180). This individualization of care exemplifies the transition towards precision medicine within sepsis treatment. Lastly, Ouyang Xiaojun et al. delineated the incidence and risk factors related to sepsis-associated acute kidney injury in pediatric patients, suggestinging actionable variables linked to poor outcomes, thereby enriching the clinical toolkit available for managing septic conditions in vulnerable populations (181).

Thus, the conglomeration of these studies epitomizes a pivotal shift toward precision-based diagnostics, innovative therapeutic interventions, and enhanced prognostic capabilities. The collective insights advocate for the integration of multidisciplinary approaches that encompass molecular biology, bioinformatics, clinical economics, and personalized medicine into the ongoing battle against sepsis, reaffirming the necessity for continuous research and development in this critical area of healthcare.

## 7 Future research directions

Future research in sepsis management is increasingly focusing on innovative methodologies and personalized approaches to improve patient outcomes. One promising direction is the integration of multi-omics data, which combines genomic, transcriptomic, proteomic, and metabolomic information to provide a comprehensive understanding of the biological processes underlying sepsis (182, 183). This integrative analysis can enhance the stratification of patients based on their unique biological profiles, potentially leading to tailored therapeutic interventions. Recent advancements in computational techniques, such as machine learning and hypergraph convolutional networks, facilitate the integration of multi-omics data, enabling researchers to derive meaningful insights from complex datasets. However, challenges remain in terms of data standardization, high dimensionality, and the need for robust analytical frameworks to ensure accurate interpretations (184). As the field progresses, future studies should prioritize the development of integrative multi-omics clustering methods that can effectively identify patient subgroups and suggest targeted therapies, thereby advancing precision medicine in sepsis management.

### 7.1 Potential of integrative multi-omics analysis

Integrative multi-omics analysis holds significant potential for elucidating the complex pathophysiological mechanisms underlying sepsis. By systematically integrating data from multiple omics domains, researchers can delineate molecular pathogenesis, identify novel candidate biomarkers, and uncover mechanistically relevant therapeutic targets. For example, integrating metabolomics with transcriptomic and proteomic data may characterize metabolic perturbations in sepsis, enabling earlier detection of disease progression and risk-stratified prognostic models (185). Emerging evidence indicates that multi-omics frameworks improve discriminatory power in early cancer detection, highlighting the translational potential of these approaches for sepsis phenotyping. Systematic integration of multi-dimensional datasets enables mechanism-centric disease characterization while prioritizing biomarker candidates for tailored therapeutic interventions (186). However, realizing the full clinical potential of multi-omics requires developing standardized analytical pipelines and rigorous validation frameworks to ensure biological interpretability and bedside applicability.

While multi-omics platforms and machine learning models offer substantial diagnostic and prognostic potential, they come with significant costs, particularly in terms of infrastructure, data analysis, and ongoing validation. In contrast, biomarkers such as procalcitonin (PCT), which have been widely validated for guiding antimicrobial stewardship, are cost-effective and readily available, offering clear cost-saving benefits. PCT-guided management has been shown to reduce antibiotic use and hospital length of stay, contributing to lower healthcare costs without compromising patient outcomes. This comparison underscores the need for a balanced approach, where the integration of cutting-edge technologies like multi-omics should be weighed against their economic feasibility, especially in resource-constrained settings.

### 7.2 Application of personalized medicine in sepsis management

Current limitations in sepsis management stem from uniform therapeutic algorithms that fail to account for interpatient heterogeneity in host-pathogen interactions and comorbid disease trajectories. Such non-stratified management paradigms neglect critical biological variables including immune endotype diversity, genetic susceptibility loci, and comorbidity-driven pathophysiological states (187). These limitations underscore the necessity for mechanistically informed stratification frameworks to optimize therapeutic precision. Precision medicine approaches leveraging multi-omic profiling and computational biology present transformative opportunities for endotype-specific sepsis care (188).

This paradigm employs multi-omic profiling (genomic, proteomic, metabolomic) to decode host-response signatures and pathogen virulence determinants (187). Emerging research identifies circulating microRNAs as dynamic risk stratification biomarkers, enabling time-sensitive therapeutic modulation (189). Systematic mapping of gene regulatory networks and molecular interactomes reveals therapeutically actionable nodes for personalized targeting (190). Integrative analysis of multi-omic datasets facilitates computational subphenotyping, guiding mechanism-targeted therapies with demonstrated mortality reduction in recent trials (191). Concurrently, AI-driven predictive modeling extracts clinically relevant patterns from high-dimensional biomarker matrices, enabling real-time treatment response forecasting (192). Translating these advances into practice necessitates overcoming implementation challenges through multicenter validation studies and evidence-based implementation roadmaps.

## 8 Conclusion

Research into biomarkers for early sepsis diagnosis and monitoring shows promise, marking a crucial change in handling this serious condition. While incorporating inflammation factors, metabolic markers, and gene expression profiles into clinical practice has the potential to enhance early detection and support timely intervention, it is equally important to acknowledge the immediate clinical applicability of cost-effective biomarkers for rapid triage and decision-making.

This review emphasizes the complementary roles of bedside biomarkers, such as procalcitonin and soluble CD14 subtype, which are already proven to enhance early diagnosis and antibiotic stewardship, particularly in resource-limited settings. Additionally, we explore the potential of integrating advanced omics technologies for precision phenotyping, especially in complex cases where traditional biomarkers may not suffice.

In line with current needs, we propose a “Hybrid Model” for the future of sepsis management: an integrated approach that combines cost-effective bedside biomarkers with advanced omics technologies. This model ensures rapid, actionable insights from easily accessible biomarkers for initial patient assessment, while utilizing omics for more precise, personalized care in challenging cases. This hybrid strategy allows for a scalable, cost-effective, and clinically applicable approach to sepsis management, which can be tailored to diverse healthcare systems worldwide.

Moving forward, it is essential to develop combined panels of point-of-care (POC) biomarkers alongside quick microbiological diagnostics, enabling healthcare providers to make fast, informed decisions. While omics technologies offer great promise for sepsis stratification, the immediate relevance of POC diagnostics and stewardship-guided biomarkers cannot be overstated, especially in environments where timely interventions are critical. The success of this hybrid model will hinge on validating these biomarkers in clinical trials, refining them through real-world application, and ensuring their integration into scalable sepsis pathways applicable across diverse healthcare systems.

Ultimately, bridging the gap between research and clinical application requires a concerted effort to balance innovative technologies with clinically validated, cost-effective tools. By combining cutting-edge omics with accessible diagnostics, we can pave the way toward revolutionizing sepsis care, moving toward a future where early detection, personalized treatment, and improved patient outcomes are the norm.

## Glossary

CRP: C-reactive protein
PCT: procalcitonin
IL-6: interleukin-6
nCD64: neutrophil CD64
TyG: triglyceride-glucose
COPD: chronic obstructive pulmonary disease
aHR: adjusted hazard ratios
CI: coagulation index
IL-10: interleukin-10
TNF-α: tumor necrosis factor-alpha
CARS: compensatory anti-inflammatory response syndrome
SII: systemic immune-inflammation index
CK-MB: creatine kinase isoenzyme MB
FFA: free fatty acid
SIMD: sepsis-induced myocardial dysfunction
CT: computed tomography
MRI: magnetic resonance imaging
POCUS: point-of-care ultrasound
AKI: acute kidney injury
miRNAs: microRNAs
lncRNAs: long non-coding RNAs
circRNAs: circular RNAs
HOTTIP: HOXA distal transcript antisense RNA
ALL: acute lymphoblastic leukemia
sCD137: soluble CD137
NLR: Neutrophil-to-Lymphocyte Ratio
PLR: Platelet-to-Neutrophil Ratio
SI-AKI: sepsis-induced acute kidney injury
cfDNA: cell-free DNA
PRM: Parallel Reaction Monitoring
GBDT: gradient boosting decision tree
mNGS: metagenomic next-generation sequencing
CCDSSs: computerized clinician decision support systems
PBMCs: peripheral blood mononuclear cells
SBO: skull base osteomyelitis
SALI: sepsis-induced acute lung injury
OAS3: oligoadenylate synthetase 3
qSOFA: quick Sequential Organ Failure Assessment
PTX3: pentraxin 3
PGJXD: Po-Ge-Jiu-Xin decoction
CLP: cecal ligation and puncture
ICER: Incremental Cost-Effectiveness Ratio
MDW: monocyte distribution width
NRGs: neutrophil-related genes.

## References

[1] Timbrook TT Morton JB McConeghy KW Caffrey AR Mylonakis E LaPlante KL . The Effect of Molecular Rapid Diagnostic Testing on Clinical Outcomes in Bloodstream Infections: A Systematic Review and Meta-analysis. Clin Infect Dis 2017. doi: 10.1093/cid/ciw649. Epub. (2016) 64:15–23. doi: 10.1093/cid/ciw649, PMID: 27678085

[2] Ma L Zhang H Yin YL Guo WZ Ma YQ Wang YB . Role of interleukin-6 to differentiate sepsis from non-infectious systemic inflammatory response syndrome. Cytokine. (2016) 88:126–35. doi: 10.1016/j.cyto.2016.08.033, PMID: 27599258

[3] Groeneveld NS Olie SE Visser DH Snoek L van de Beek D Brouwer MC . Cerebrospinal fluid inflammatory markers to differentiate between neonatal bacterial meningitis and sepsis: A prospective study of diagnostic accuracy. Int J Infect Dis. (2024) 142:106970. doi: 10.1016/j.ijid.2024.02.013, PMID: 38395221

[4] Pierrakos C Vincent JL . Sepsis biomarkers: a review. Crit Care. (2010) 14:R15. doi: 10.1186/cc8872, PMID: 20144219 PMC2875530

[5] Wacker C Prkno A Brunkhorst FM Schlattmann P . Procalcitonin as a diagnostic marker for sepsis: a systematic review and meta-analysis. Lancet Infect Dis. (2013) 13:426–35. doi: 10.1016/S1473-3099(12)70323-7, PMID: 23375419

[6] Dark P Hossain A McAuley DF Brealey D Carlson G Clayton JC . Biomarker-Guided Antibiotic Duration for Hospitalized Patients With Suspected Sepsis: The ADAPT-Sepsis Randomized Clinical Trial. JAMA. (2025) 333:682–93. doi: 10.1001/jama.2024.26458. Erratum in: JAMA PMC11862976 39652885

[7] Martin MD Badovinac VP Griffith TS . CD4 T Cell Responses and the Sepsis-Induced Immunoparalysis State. Front Immunol. (2020) 11:1364. doi: 10.3389/fimmu.2020.01364, PMID: 32733454 PMC7358556

[8] Paalani M Lee JW Haddad E Tonstad S . Determinants of inflammatory markers in a bi-ethnic population. Ethn Dis. (2011) 21:142–9. PMC3427005 21749016

[9] Komorowski M Green A Tatham KC Seymour C Antcliffe D . Sepsis biomarkers and diagnostic tools with a focus on machine learning. EBioMedicine. (2022) 86:104394. doi: 10.1016/j.ebiom.2022.104394, PMID: 36470834 PMC9783125

[10] Singer M Deutschman CS Seymour CW Shankar-Hari M Annane D Bauer M . The Third International Consensus Definitions for Sepsis and Septic Shock (Sepsis-3). JAMA. (2016) 315:801–10. doi: 10.1001/jama.2016.0287, PMID: 26903338 PMC4968574

[11] Rhodes A Evans LE Alhazzani W Levy MM Antonelli M Ferrer R . Surviving Sepsis Campaign: International Guidelines for Management of Sepsis and Septic Shock: 2016. Intensive Care Med. (2017) 43:304–77. doi: 10.1007/s00134-017-4683-6, PMID: 28101605

[12] Wang X Cui X Fan H Hu T . Elevated Triglyceride-Glucose (TyG) Index Predicts Poor Clinical Outcomes in Critically Ill AECOPD Patients: A Retrospective Study. Int J Chron Obstruct Pulmon Dis. (2024) 19:2217–28. doi: 10.2147/COPD.S477268, PMID: 39371919 PMC11453155

[13] Getsina M Chernevskaya E Beloborodova N Golovnya E Polyakov P Kushlinskii N . Features of Metabolites and Biomarkers in Inflammatory and Infectious Complications of Childhood Cancers. Biomedicines. (2024) :2101. doi: 10.3390/biomedicines12092101, PMID: 39335614 PMC11429149

[14] Liu DX Didier PJ Plauche G Pahar B . Septicemia in an Indian Rhesus Macaque (Macaca mulatta) associated with Providencia stuartii. J Med Primatol. (2016) 45:330–2. doi: 10.1111/jmp.12230, PMID: 27466784 PMC5274604

[15] Morley PT . Early Fluid Management in Sepsis: Yes. Crit Care Med. (2018) 46:327–8. doi: 10.1097/CCM.0000000000002880, PMID: 29337792

[16] Petel DS Isabel S Lee KS Ting JY Kaufman DA Sanchez PJ . Empiric antibiotic prescribing practices for gram-positive coverage of late-onset sepsis in neonatal intensive care units in North America. Infect Control Hosp Epidemiol. (2024) 46:1–3. doi: 10.1017/ice.2024.176. Epub ahead of print PMC11717478 39506373

[17] Evans L Rhodes A Alhazzani W Antonelli M Coopersmith CM French C . Surviving sepsis campaign: international guidelines for management of sepsis and septic shock 2021. Intensive Care Med. (2021) 47:1181–247. doi: 10.1007/s00134-021-06506-y, PMID: 34599691 PMC8486643

[18] Basile-Filho A Lago AF Menegueti MG Nicolini EA Rodrigues LAB Nunes RS . SOFA, SAPS 3, C-reactive protein/albumin ratio, and lactate to predict mortality of surgical critically ill patients: A retrospective cohort study. Med (Baltimore). (2019) 98:e16204. doi: 10.1097/MD.0000000000016204, PMID: 31261567 PMC6617482

[19] Zhang YY Ning BT . Signaling pathways and intervention therapies in sepsis. Signal Transduct Target Ther. (2021) 6:407. doi: 10.1038/s41392-021-00816-9, PMID: 34824200 PMC8613465

[20] He RR Yue GL Dong ML Wang JQ Cheng C . Sepsis Biomarkers: Advancements and Clinical Applications-A Narrative Review. Int J Mol Sci. (2024) 25:9010. doi: 10.3390/ijms25169010, PMID: 39201697 PMC11354379

[21] Wirz Y Meier MA Bouadma L Luyt CE Wolff M Chastre J . Effect of procalcitonin-guided antibiotic treatment on clinical outcomes in intensive care unit patients with infection and sepsis patients: a patient-level meta-analysis of randomized trials. Crit Care. (2018) 22:191. doi: 10.1186/s13054-018-2125-7, PMID: 30111341 PMC6092799

[22] Kyriazopoulou E Liaskou-Antoniou L Adamis G Panagaki A Melachroinopoulos N Drakou E . Procalcitonin to Reduce Long-Term Infection-associated Adverse Events in Sepsis. A Randomized Trial. Am J Respir Crit Care Med. (2021) 203:202–10. doi: 10.1164/rccm.202004-1201OC, PMID: 32757963 PMC7874409

[23] Pierrakos C Velissaris D Bisdorff M Marshall JC Vincent JL . Biomarkers of sepsis: time for a reappraisal. Crit Care. (2020) 24:287. doi: 10.1186/s13054-020-02993-5, PMID: 32503670 PMC7273821

[24] Hattori T Nishiyama H Kato H Ikegami S Nagayama M Asami S . Clinical value of procalcitonin for patients with suspected bloodstream infection. Am J Clin Pathol. (2014) 141:43–51. doi: 10.1309/AJCP4GV7ZFDTANGC, PMID: 24343736

[25] Park JH Kim DH Jang HR Kim MJ Jung SH Lee JE . Clinical relevance of procalcitonin and C-reactive protein as infection markers in renal impairment: a cross-sectional study. Crit Care. (2014) 18:640. doi: 10.1186/s13054-014-0640-8, PMID: 25407928 PMC4279682

[26] Velissaris D Zareifopoulos N Karamouzos V Karanikolas E Pierrakos C Koniari I . Presepsin as a Diagnostic and Prognostic Biomarker in Sepsis. Cureus. (2021) 13:e15019. doi: 10.7759/cureus.15019, PMID: 34150378 PMC8202808

[27] Paraskevas T Chourpiliadi C Demiri S Micahilides C Karanikolas E Lagadinou M . Presepsin in the diagnosis of sepsis. Clin Chim Acta. (2023) 550:117588. doi: 10.1016/j.cca.2023.117588, PMID: 37813329

[28] Angeletti S Spoto S Fogolari M Cortigiani M Fioravanti M De Florio L . Diagnostic and prognostic role of procalcitonin (PCT) and MR-pro-Adrenomedullin (MR-proADM) in bacterial infections. APMIS. (2015) 123:740–8. doi: 10.1111/apm.12406, PMID: 26058482

[29] Önal U Valenzuela-Sánchez F Vandana KE Rello J . Mid-Regional Pro-Adrenomedullin (MR-proADM) as a Biomarker for Sepsis and Septic Shock: Narrative Review. Healthcare (Basel). (2018) 6:110. doi: 10.3390/healthcare6030110, PMID: 30177659 PMC6164535

[30] Chen W Guo W Li Y Chen M . Integrative analysis of metabolomics and transcriptomics to uncover biomarkers in sepsis. Sci Rep. (2024) 14:9676. doi: 10.1038/s41598-024-59400-0, PMID: 38678059 PMC11055861

[31] Peng J Xiang Y Liu G Ling S Li F . The early prognostic value of the 1-4-day BCM/PA trend after admission in neurocritical patients. Sci Rep. (2024) 14:21802. doi: 10.1038/s41598-024-72142-3, PMID: 39294206 PMC11410815

[32] Hasibuan BS Dasatjipta G Lubis BM Sanny S . Role of neutrophil-to-lymphocyte ratio and platelet-to-lymphocyte ratio in diagnosing neonatal sepsis. Narra J. (2024) 4:e763. doi: 10.52225/narra.v4i2.763, PMID: 39280270 PMC11391992

[33] Fang X Fu W Xu L Qiu Y . Analysis of the diagnostic value of coagulation markers and coagulation function indices on the occurrence of DIC in sepsis and its prognosis. Allergol Immunopathol (Madr). (2024) 52:65–72. doi: 10.15586/aei.v52i5.1119, PMID: 39278853

[34] Keller D Mester P Räth U Krautbauer S Schmid S Greifenberg V . Calprotectin, a Promising Serological Biomarker for the Early Diagnosis of Superinfections with Multidrug-Resistant Bacteria in Patients with COVID-19. Int J Mol Sci. (2024) 25:9294. doi: 10.3390/ijms25179294, PMID: 39273246 PMC11394900

[35] Garbern SC Mamun GMS Shaima SN Hakim N Wegerich S Alla S . A novel digital health approach to improving global pediatric sepsis care in Bangladesh using wearable technology and machine learning. PloS Digit Health. (2024) 3:e0000634. doi: 10.1371/journal.pdig.0000634, PMID: 39475844 PMC11524492

[36] Khuchua E Didbaridze T Ormotsadze G Sanikidze T Pachkoria E Ratiani L . Evaluating the Diagnostic and Prognostic Value of Interleukin-6 (IL-6) and Soluble Triggering Receptor Expressed on Myeloid Cells-1 (sTREM-1) in Systemic Inflammatory Response Syndrome (SIRS) and Sepsis in Adults. Cureus. (2024) 16:e73310. doi: 10.7759/cureus.73310, PMID: 39655134 PMC11626217

[37] Song J Park DW Moon S Cho HJ Park JH Seok H . Diagnostic and prognostic value of interleukin-6, pentraxin 3, and procalcitonin levels among sepsis and septic shock patients: a prospective controlled study according to the Sepsis-3 definitions. BMC Infect Dis. (2019) 19:968. doi: 10.1186/s12879-019-4618-7, PMID: 31718563 PMC6852730

[38] Scheer C Fuchs C Rehberg S . Biomarkers in Severe Sepsis and Septic Shock: Just Listen to the Heart? Crit Care Med. (2016) 44:849–50. doi: 10.1097/CCM.0000000000001507, PMID: 26974450

[39] Molano-Franco D Arevalo-Rodriguez I Muriel A Del Campo-Albendea L Fernández-García S Alvarez-Méndez A . Basal procalcitonin, C-reactive protein, interleukin-6, and presepsin for prediction of mortality in critically ill septic patients: a systematic review and meta-analysis. Diagn Progn Res. (2023) 7:15. doi: 10.1186/s41512-023-00152-2, PMID: 37537680 PMC10399020

[40] Xie Y Zhuang D Chen H Zou S Chen W Chen Y . 28-day sepsis mortality prediction model from combined serial interleukin-6, lactate, and procalcitonin measurements: a retrospective cohort study. Eur J Clin Microbiol Infect Dis. (2023) 42:77–85. doi: 10.1007/s10096-022-04517-1, PMID: 36383295 PMC9816294

[41] Chen L Lu Y Zhao L Hu L Qiu Q Zhang Z . Corrigendum to "Curcumin attenuates sepsis-induced acute organ dysfunction by preventing inflammation and enhancing the suppressive function of Tregs" [Int. Immunopharmacol. 61 (2018) 1-7]. Int Immunopharmacol. (2024) 137:112471. doi: 10.1016/j.intimp.2024.112471. Erratum for: Int Immunopharmacol. 2018 61:1-7. doi: 10.1016/j.intimp.2018.04.041. PMID: 38879417

[42] Numerof RP Asadullah K . Cytokine and anti-cytokine therapies for psoriasis and atopic dermatitis. BioDrugs. (2006) 20:93–103. doi: 10.2165/00063030-200620020-00004, PMID: 16626167

[43] Min J Zhao Y Lv C Hu H . Red blood cell count in cerebrospinal fluid was correlated with inflammatory markers on the seventh postoperative day and all associated with the outcome of aneurysmal subarachnoid hemorrhage patients. Front Med (Lausanne). (2024) 11:1408126. doi: 10.3389/fmed.2024.1408126, PMID: 38860207 PMC11163054

[44] Kopp JB Anders HJ Susztak K Podestà MA Remuzzi G Hildebrandt F . Podocytopathies. Nat Rev Dis Primers. (2020) 6:68. doi: 10.1038/s41572-020-0196-7, PMID: 32792490 PMC8162925

[45] Huang G Yang W Zhao X Bai Y Jiang X Liu J . Analysis of Prognostic Risk Factors of Sepsis Patients With Myocardial Injury: Six-month Survival Outcome. Altern Ther Health Med. (2023) 29:744–9. 37708545

[46] Luo ZL Ren JD Huang Z Wang T Xiang K Cheng L . The Role of Exogenous Hydrogen Sulfide in Free Fatty Acids Induced Inflammation in Macrophages. Cell Physiol Biochem. (2017) 42:1635–44. doi: 10.1159/000479405, PMID: 28738323

[47] Chen H Ding Y Chen W Feng Y Shi G . Glibenclamide alleviates inflammation in oleic acid model of acute lung injury through NLRP3 inflammasome signaling pathway. Drug Des Devel Ther. (2019) 13:1545–54. doi: 10.2147/DDDT.S196040, PMID: 31123394 PMC6511253

[48] Yu H Yang Q Qian Y Luo S Kong T Yang G . A positive correlation between serum lactate dehydrogenase level and in-hospital mortality in ICU sepsis patients: evidence from two large databases. Eur J Med Res. (2024) 29:525. doi: 10.1186/s40001-024-02071-4, PMID: 39487549 PMC11531135

[49] Dundar MA Ceran E Akyildiz BN . Prognostic and diagnostic utility of pancreatic stone protein in pediatric sepsis and mortality. Turk J Med Sci. (2024) 54:744–51. doi: 10.55730/1300-0144.5844, PMID: 39295616 PMC11407330

[50] Tsakiroglou M Evans A Pirmohamed M . Leveraging transcriptomics for precision diagnosis: Lessons learned from cancer and sepsis. Front Genet. (2023) 14:1100352. doi: 10.3389/fgene.2023.1100352, PMID: 36968610 PMC10036914

[51] Zhang WY Chen ZH An XX Li H Zhang HL Wu SJ . Analysis and validation of diagnostic biomarkers and immune cell infiltration characteristics in pediatric sepsis by integrating bioinformatics and machine learning. World J Pediatr. (2023) 19:1094–103. doi: 10.1007/s12519-023-00717-7, PMID: 37115484 PMC10533616

[52] Yang H Feng L Jiang Z Wu X Zeng K . Amlexanox reduces new-onset atrial fibrillation risk in sepsis by downregulating S100A12: a Mendelian randomization study. Front Cardiovasc Med. (2024) 11:1401314. doi: 10.3389/fcvm.2024.1401314, PMID: 39444551 PMC11496243

[53] Kong C Zhu Y Xie X Wu J Qian M . Six potential biomarkers in septic shock: a deep bioinformatics and prospective observational study. Front Immunol. (2023) 14:1184700. doi: 10.3389/fimmu.2023.1184700, PMID: 37359526 PMC10285480

[54] Santacroce E D'Angerio M Ciobanu AL Masini L Lo Tartaro D Coloretti I . Advances and Challenges in Sepsis Management: Modern Tools and Future Directions. Cells. (2024) 13:439. doi: 10.3390/cells13050439, PMID: 38474403 PMC10931424

[55] Evans L Rhodes A Alhazzani W Antonelli M Coopersmith CM French C . Executive Summary: Surviving Sepsis Campaign: International Guidelines for the Management of Sepsis and Septic Shock 2021. Crit Care Med. (2021) 49:1974–82. doi: 10.1097/CCM.0000000000005357. Erratum in: Crit Care Med 34643578

[56] Gu Y Li Z Li H Yi X Liu X Zhang Y . Exploring the efficacious constituents and underlying mechanisms of sini decoction for sepsis treatment through network pharmacology and multi-omics. Phytomedicine. (2024) 123:155212. doi: 10.1016/j.phymed.2023.155212, PMID: 38029626

[57] Ding X Liang W Xia H Liu Y Liu S Xia X . Analysis of Immune and Prognostic-Related lncRNA PRKCQ-AS1 for Predicting Prognosis and Regulating Effect in Sepsis. J Inflammation Res. (2024) 17:279–99. doi: 10.2147/JIR.S433057, PMID: 38229689 PMC10790647

[58] Liu Y Qu HQ Chang X Tian L Qu J Glessner J . Machine Learning Reduced Gene/Non-Coding RNA Features That Classify Schizophrenia Patients Accurately and Highlight Insightful Gene Clusters. Int J Mol Sci. (2021) 22:3364. doi: 10.3390/ijms22073364, PMID: 33805976 PMC8037538

[59] Cummings MJ Lutwama JJ Tomoiaga AS Zhao M Owor N Lu X . Identification of transcriptomic sepsis endotypes in sub-Saharan Africa: derivation, validation, and global alignment in two Ugandan cohorts. Intensive Care Med. (2025) 51:1573–86. doi: 10.1007/s00134-025-08047-0, PMID: 40728637 PMC12405036

[60] Gofton TE Young GB . Sepsis-associated encephalopathy. Nat Rev Neurol. (2012) 8:557–66. doi: 10.1038/nrneurol.2012.183, PMID: 22986430

[61] Polyzogopoulou E Velliou M Verras C Ventoulis I Parissis J Osterwalder J . Point-of-Care Ultrasound: A Multimodal Tool for the Management of Sepsis in the Emergency Department. Medicina (Kaunas). (2023) 59:1180. doi: 10.3390/medicina59061180, PMID: 37374384 PMC10303071

[62] Sweeney DA Wiley BM . Integrated Multiorgan Bedside Ultrasound for the Diagnosis and Management of Sepsis and Septic Shock. Semin Respir Crit Care Med. (2021) 42:641–9. doi: 10.1055/s-0041-1733896, PMID: 34544181

[63] Verras C Ventoulis I Bezati S Matsiras D Parissis J Polyzogopoulou E . Point of Care Ultrasonography for the Septic Patient in the Emergency Department: A Literature Review. J Clin Med. (2023) 12:1105. doi: 10.3390/jcm12031105, PMID: 36769753 PMC9917776

[64] Trapnell BC . A novel biomarker-guided immunomodulatory approach for the therapy of sepsis. Am J Respir Crit Care Med. (2009) 180:585–6. doi: 10.1164/rccm.200907-1095ED, PMID: 19762591

[65] Li G Zhang W Gu W . The Relationship Between Soluble Interleukin-17 Receptor Levels and CD3-Positive T Cells and Lymphocytes in Patients with Sepsis and Their Predictive Clinical Significance. J Inflammation Res. (2024) 17:7543–50. doi: 10.2147/JIR.S479310, PMID: 39464344 PMC11505382

[66] Ji D Li J Liu A Ye R Zhang S Gao L . Predictive Value of Combined Detection of Serum LGALS3BP and GDF-15 for the Prognosis of ICU Sepsis Patients. Infect Drug Resist. (2024) 17:4417–26. doi: 10.2147/IDR.S468298, PMID: 39431211 PMC11488509

[67] Luka S Golea A Tat RM Lupan Mureșan EM Voicescu GT Vesa C . Biomarkers as Predictors of Mortality in Sepsis and Septic Shock for Patients Admitted to Emergency Department: Who Is the Winner? A Prospective Study. J Clin Med. (2024) 13:5678. doi: 10.3390/jcm13195678, PMID: 39407738 PMC11477125

[68] Lou J Xiang Z Zhu X Fan Y Song J Cui S . A retrospective study utilized MIMIC-IV database to explore the potential association between triglyceride-glucose index and mortality in critically ill patients with sepsis. Sci Rep. (2024) 14:24081. doi: 10.1038/s41598-024-75050-8, PMID: 39402158 PMC11473526

[69] Xin Y Liu Y Liu L Wang X Wang D Song Y . Dynamic changes in the real-time glomerular filtration rate and kidney injury markers in different acute kidney injury models. J Transl Med. (2024) 22:857. doi: 10.1186/s12967-024-05667-w, PMID: 39334187 PMC11430329

[70] Jabornisky R Kuppermann N Gonzalez-Dambrauskas S . Transitioning From SIRS to Phoenix With the Updated Pediatric Sepsis Criteria-The Difficult Task of Simplifying the Complex. JAMA. (2024) 331:650–1. doi: 10.1001/jama.2023.27988, PMID: 38245901

[71] Bonkat G Cai T Veeratterapillay R Bruyère F Bartoletti R Pilatz A . Management of Urosepsis in 2018. Eur Urol Focus. (2019) 5:5–9. doi: 10.1016/j.euf.2018.11.003, PMID: 30448051

[72] Karampela I Fragkou PC . Future Perspectives in the Diagnosis and Treatment of Sepsis and Septic Shock. Medicina (Kaunas). (2022) 58:844. doi: 10.3390/medicina58070844, PMID: 35888563 PMC9323821

[73] Lam SM Lau AC Lam RP Yan WW . Clinical management of sepsis. Hong Kong Med J. (2017) 23:296–305. doi: 10.12809/hkmj165057, PMID: 28572520

[74] Xu J Tao L Jiang L Lai J Hu J Tang Z . Moderate Hypothermia Alleviates Sepsis-Associated Acute Lung Injury by Suppressing Ferroptosis Induced by Excessive Inflammation and Oxidative Stress via the Keap1/GSK3β/Nrf2/GPX4 Signaling Pathway. J Inflammation Res. (2024) 17:7687–704. doi: 10.2147/JIR.S491885, PMID: 39498104 PMC11533192

[75] Yuan J Li M Chang X Chen Y Dong C . [Clinical value of non-coding RNA molecular markers for sepsis]. Zhonghua Wei Zhong Bing Ji Jiu Yi Xue. (2019) 31:650–3. doi: 10.3760/cma.j.issn.2095-4352.2019.05.026, PMID: 31198158

[76] Chen Y Jing H Tang S Liu P Cheng Y Fan Y . Non-Coding RNAs in Sepsis-Associated Acute Kidney Injury. Front Physiol. (2022) 13:830924. doi: 10.3389/fphys.2022.830924, PMID: 35464083 PMC9024145

[77] Beltrán-García J Osca-Verdegal R Nácher-Sendra E Cardona-Monzonís A Sanchis-Gomar F Carbonell N . Role of non-coding RNAs as biomarkers of deleterious cardiovascular effects in sepsis. Prog Cardiovasc Dis. (2021) 68:70–7. doi: 10.1016/j.pcad.2021.07.005, PMID: 34265333

[78] Shi W Zhu W Yu J Shi Y Zhao Y . LncRNA HOTTIP as a diagnostic biomarker for acute respiratory distress syndrome in patients with sepsis and to predict the short-term clinical outcome: a case-control study. BMC Anesthesiol. (2024) 24:30. doi: 10.1186/s12871-024-02405-z, PMID: 38238652 PMC10795278

[79] Fan H Shao H Gao X . Long Non-Coding RNA HOTTIP is Elevated in Patients with Sepsis and Promotes Cardiac Dysfunction. Immunol Invest. (2022) 51:2086–96. doi: 10.1080/08820139.2022.2107932, PMID: 35921152

[80] Petejova N Martinek A Zadrazil J Kanova M Klementa V Sigutova R . Acute Kidney Injury in Septic Patients Treated by Selected Nephrotoxic Antibiotic Agents-Pathophysiology and Biomarkers-A Review. Int J Mol Sci. (2020) 21:7115. doi: 10.3390/ijms21197115, PMID: 32993185 PMC7583998

[81] Ju JK Han WN Shi CL Long non-coding RNA . (lncRNA) plasmacytoma variant translocation 1 gene (PVT1) modulates the proliferation and apoptosis of acute lymphoblastic leukemia cells by sponging miR-486-5p. Bioengineered. (2022) 13:4587–97. doi: 10.1080/21655979.2022.2031405, PMID: 35152842 PMC8973597

[82] Dhudasia MB Benitz WE Flannery DD Christ L Rub D Remaschi G . Diagnostic Performance and Patient Outcomes With C-Reactive Protein Use in Early-Onset Sepsis Evaluations. J Pediatr. (2023) 256:98–104. doi: 10.1016/j.jpeds.2022.12.007, PMID: 36529283 PMC10164676

[83] Liu Y Gao Y Liang B Liang Z . The prognostic value of C-reactive protein to albumin ratio in patients with sepsis: a systematic review and meta-analysis. Aging Male. (2023) 26:2261540. doi: 10.1080/13685538.2023.2261540, PMID: 37752726

[84] Silvinato A Lucato Dos Santos C Amorim E Floriano I Paciência LEM Tristão LS . C-reactive protein in adult sepsis: systematic review and meta-analysis. Clinics (Sao Paulo). (2025) 81:100848. doi: 10.1016/j.clinsp.2025.100848, PMID: 41429071 PMC12795679

[85] Chambliss AB Patel K Colón-Franco JM Hayden J Katz SE Minejima E . AACC Guidance Document on the Clinical Use of Procalcitonin. J Appl Lab Med. (2023) 8:598–634. doi: 10.1093/jalm/jfad007, PMID: 37140163

[86] Oczkowski S Alshamsi F Belley-Cote E Centofanti JE Hylander Møller M Nunnaly ME . Surviving Sepsis Campaign Guidelines 2021: highlights for the practicing clinician. Pol Arch Intern Med. (2022) 132:16290. doi: 10.20452/pamw.16290, PMID: 35791800

[87] Zaki HA Bensliman S Bashir K Iftikhar H Fayed MH Salem W . Accuracy of procalcitonin for diagnosing sepsis in adult patients admitted to the emergency department: a systematic review and meta-analysis. Syst Rev. (2024) 13:37. doi: 10.1186/s13643-023-02432-w, PMID: 38254218 PMC10802075

[88] Molano Franco D Arevalo-Rodriguez I Roqué I Figuls M Montero Oleas NG Nuvials X Zamora J . Plasma interleukin-6 concentration for the diagnosis of sepsis in critically ill adults. Cochrane Database Syst Rev. (2019) 4:CD011811. doi: 10.1002/14651858.CD011811.pub2, PMID: 31038735 PMC6490303

[89] Varga NI Bagiu IC Vulcanescu DD Lazureanu V Turaiche M Rosca O . IL-6 Baseline Values and Dynamic Changes in Predicting Sepsis Mortality: A Systematic Review and Meta-Analysis. Biomolecules. (2025) 15:407. doi: 10.3390/biom15030407, PMID: 40149943 PMC11940105

[90] Barichello T Generoso JS Singer M Dal-Pizzol F . Biomarkers for sepsis: more than just fever and leukocytosis-a narrative review. Crit Care. (2022) 26:14. doi: 10.1186/s13054-021-03862-5, PMID: 34991675 PMC8740483

[91] Candelli M Sacco Fernandez M Rozzi G Sodero G Piccioni A Pignataro G . The Interleukin Network in Sepsis: From Cytokine Storm to Clinical Applications. Diagnostics (Basel). (2025) 15:2927. doi: 10.3390/diagnostics15222927, PMID: 41300951 PMC12651451

[92] Saraiva M Vieira P O'Garra A . Biology and therapeutic potential of interleukin-10. J Exp Med. (2020) 217:e20190418. doi: 10.1084/jem.20190418, PMID: 31611251 PMC7037253

[93] Zhang X Zhang Y Yuan S Zhang J . The potential immunological mechanisms of sepsis. Front Immunol. (2024) 15:1434688. doi: 10.3389/fimmu.2024.1434688, PMID: 39040114 PMC11260823

[94] Georgescu AM Banescu C Azamfirei R Hutanu A Moldovan V Badea I . Evaluation of TNF-α genetic polymorphisms as predictors for sepsis susceptibility and progression. BMC Infect Dis. (2020) 20:221. doi: 10.1186/s12879-020-4910-6, PMID: 32171247 PMC7071754

[95] Gharamti AA Samara O Monzon A Montalbano G Scherger S DeSanto K . Proinflammatory cytokines levels in sepsis and healthy volunteers, and tumor necrosis factor-alpha associated sepsis mortality: A systematic review and meta-analysis. Cytokine. (2022) 158:156006. doi: 10.1016/j.cyto.2022.156006, PMID: 36044827

[96] Hung SK Lan HM Han ST Wu CC Chen KF . Current Evidence and Limitation of Biomarkers for Detecting Sepsis and Systemic Infection. Biomedicines. (2020) 8:494. doi: 10.3390/biomedicines8110494, PMID: 33198109 PMC7697922

[97] Pham HM Nguyen DLM Duong MC Phan XT Tran LT Trang DHT . Neutrophil CD64-a prognostic marker of sepsis in intensive care unit: a prospective cohort study. Front Med (Lausanne). (2023), 10:1251221. doi: 10.3389/fmed.2023.1251221, PMID: 37746077 PMC10514672

[98] Wang X Li ZY Zeng L Zhang AQ Pan W Gu W . Neutrophil CD64 expression as a diagnostic marker for sepsis in adult patients: a meta-analysis. Crit Care. (2015) 19:245. doi: 10.1186/s13054-015-0972-z, PMID: 26059345 PMC4490738

[99] Jolly L Carrasco K Salcedo-Magguilli M Garaud JJ Lambden S van der Poll T . sTREM-1 is a specific biomarker of TREM-1 pathway activation. Cell Mol Immunol. (2021) 18:2054–6. doi: 10.1038/s41423-021-00733-5, PMID: 34282296 PMC8322270

[100] Su L Liu D Chai W Liu D Long Y . Role of sTREM-1 in predicting mortality of infection: a systematic review and meta-analysis. BMJ Open. (2016) 6:e010314. doi: 10.1136/bmjopen-2015-010314, PMID: 27178971 PMC4874109

[101] Wu Y Wang F Fan X Bao R Bo L Li J . Accuracy of plasma sTREM-1 for sepsis diagnosis in systemic inflammatory patients: a systematic review and meta-analysis. Crit Care. (2012) 16:R229. doi: 10.1186/cc11884, PMID: 23194114 PMC3672614

[102] Moura ELB Ferreira DP Pereira RW . Presepsin as a Diagnostic and Prognostic Biomarker of Sepsis-Associated Acute Kidney Injury: A Scoping Review of Clinical Evidence. J Clin Med. (2025) 14:6970. doi: 10.3390/jcm14196970, PMID: 41096051 PMC12524575

[103] Piccioni A Baroni S Rozzi G Belvederi F Leggeri S Spagnuolo F . Evaluation of Presepsin for Early Diagnosis of Sepsis in the Emergency Department. J Clin Med. (2025) 14:2480. doi: 10.3390/jcm14072480, PMID: 40217929 PMC11989492

[104] Yang HS Hur M Yi A Kim H Lee S Kim SN . Prognostic value of presepsin in adult patients with sepsis: Systematic review and meta-analysis. PloS One. (2018) 13:e0191486. doi: 10.1371/journal.pone.0191486, PMID: 29364941 PMC5783380

[105] Beltrán-García J Osca-Verdegal R Nacher-Sendra E Pallardó FV García-Giménez JL . Circular RNAs in Sepsis: Biogenesis, Function, and Clinical Significance. Cells. (2020) 9:1544. doi: 10.3390/cells9061544, PMID: 32630422 PMC7349763

[106] Qi L Yan Y Chen B Cao J Liang G Xu P . Research progress of circRNA as a biomarker of sepsis: a narrative review. Ann Transl Med. (2021) 9:720. doi: 10.21037/atm-21-1247, PMID: 33987418 PMC8106021

[107] Wei L Yang Y Wang W Xu R . Circular RNAs in the pathogenesis of sepsis and their clinical implications: A narrative review. Ann Acad Med Singap. (2022) 51:221–7. doi: 10.47102/annals-acadmedsg.2021405, PMID: 35506405

[108] El-Sisi MG Radwan SM Ali SS Mostafa MY Hamdy NM . The long antisense non-coding RNA HOXA transcript at the distal tip (LncRNA HOTTIP) in health and disease: a comprehensive review and in silico analysis. Naunyn Schmiedebergs Arch Pharmacol. (2025) 398:16537–75. doi: 10.1007/s00210-025-04372-9, PMID: 40616679 PMC12678630

[109] Wang C Liang G Shen J Kong H Wu D Huang J . Long Non-Coding RNAs as Biomarkers and Therapeutic Targets in Sepsis. Front Immunol. (2021) :722004. doi: 10.3389/fimmu.2021.722004, PMID: 34630395 PMC8492911

[110] Sun B Guo S . miR-486-5p Serves as a Diagnostic Biomarker for Sepsis and Its Predictive Value for Clinical Outcomes. J Inflammation Res. (2021) 14:3687–95. doi: 10.2147/JIR.S323433, PMID: 34354365 PMC8331108

[111] Bindayna K . MicroRNA as Sepsis Biomarkers: A Comprehensive Review. Int J Mol Sci. (2024) 25:6476. doi: 10.3390/ijms25126476, PMID: 38928179 PMC11204033

[112] Evans L . Surviving Sepsis Campaign: International Guidelines for Management of Sepsis and Septic Shock 2021. Crit Care Med. (2021) 49:e1063–143. doi: 10.1007/s00134-021-06506-y, PMID: 34605781

[113] Doganci M Eraslan Doganay G Sazak H Alagöz A Cirik MO Hoşgün D . The Utility of C-Reactive Protein, Procalcitonin, and Leukocyte Values in Predicting the Prognosis of Patients with Pneumosepsis and Septic Shock. Medicina (Kaunas). (2024) 60:1560. doi: 10.3390/medicina60101560, PMID: 39459346 PMC11509754

[114] Gao Z Wang X Wang W Kang Z Chen X . Association between neutrophil to lymphocyte ratio and the mortality of patients with sepsis: an update systematic review and meta-analysis. Front Med (Lausanne). (2025) 12:1637365. doi: 10.3389/fmed.2025.1637365, PMID: 41189878 PMC12580286

[115] Wu H Cao T Ji T Luo Y Huang J Ma K . Predictive value of the neutrophil-to-lymphocyte ratio in the prognosis and risk of death for adult sepsis patients: a meta-analysis. Front Immunol. (2024) 15:1336456. doi: 10.3389/fimmu.2024.1336456, PMID: 38562922 PMC10982325

[116] Zhang X Liu D Liu YN Wang R Xie LX . The accuracy of presepsin (sCD14-ST) for the diagnosis of sepsis in adults: a meta-analysis. Crit Care. (2015) 19:323. doi: 10.1186/s13054-015-1032-4, PMID: 26357898 PMC4566362

[117] Zheng Z Jiang L Ye L Gao Y Tang L Zhang M . The accuracy of presepsin for the diagnosis of sepsis from SIRS: a systematic review and meta-analysis. Ann Intensive Care. (2015) 5:48. doi: 10.1186/s13613-015-0089-1, PMID: 26642970 PMC4671989

[118] Huang Q Xiong H Yan P Shuai T Liu J Zhu L . The Diagnostic and Prognostic Value of suPAR in Patients with Sepsis: A Systematic Review and Meta-Analysis. Shock. (2020) 53:416–25. doi: 10.1097/SHK.0000000000001434, PMID: 31490358 PMC7069396

[119] Pregernig A Müller M Held U Beck-Schimmer B . Prediction of mortality in adult patients with sepsis using six biomarkers: a systematic review and meta-analysis. Ann Intensive Care. (2019) 9:125. doi: 10.1186/s13613-019-0600-1, PMID: 31705327 PMC6841861

[120] Pregernig A . Prediction of mortality in adult patients with sepsis using six biomarkers: a systematic review and meta-analysis. Ann Intensive Care. (2019) 9:125. doi: 10.1186/s13613-019-0600-1, PMID: 31705327 PMC6841861

[121] Valeriani E Falletta A Pastori D Porfidia A Mastroianni CM Di Bari S . Midregional-proAdrenomedullin as a prognostic tool in sepsis and septic shock: A systematic review and meta-analysis. Eur J Clin Invest. (2024) 54:e14225. doi: 10.1111/eci.14225, PMID: 38632681

[122] Zhuo M Fu S Chi Y Li X Li S Ma X . Angiopoietin-2 as a prognostic biomarker in septic adult patients: a systemic review and meta-analysis. Ann Intensive Care. (2024) 14:169. doi: 10.1186/s13613-024-01393-0, PMID: 39522088 PMC11551087

[123] Zhuo M . Angiopoietin-2 as a prognostic biomarker in septic adult patients: a systemic review and meta-analysis. Ann Intensive Care. (2024) 14:169. doi: 10.1186/s13613-024-01393-0, PMID: 39522088 PMC11551087

[124] Sun T Wang Y Wu X Cai Y Zhai T Zhan Q . Prognostic Value of Syndecan-1 in the Prediction of Sepsis-Related Complications and Mortality: A Meta-Analysis. Front Public Health. (2022) 10:870065. doi: 10.3389/fpubh.2022.870065, PMID: 35480580 PMC9035829

[125] Iba T Maier CL Helms J Ferrer R Thachil J Levy JH . Managing sepsis and septic shock in an endothelial glycocalyx-friendly way: from the viewpoint of surviving sepsis campaign guidelines. Ann Intensive Care. (2024) 14:64. doi: 10.1186/s13613-024-01301-6, PMID: 38658435 PMC11043313

[126] Denning NL Aziz M Gurien SD Wang P . DAMPs and NETs in Sepsis. Front Immunol. (2019) 10:2536. doi: 10.3389/fimmu.2019.02536, PMID: 31736963 PMC6831555

[127] Hong Q Zhu S Yu Y Ren Y Jin L Wang H . The emerging role of mtDNA release in sepsis: Current evidence and potential therapeutic targets. J Cell Physiol. (2024) 239:e31331. doi: 10.1002/jcp.31331, PMID: 38888012

[128] van der Pol Y Moldovan N Ramaker J Bootsma S Lenos KJ Vermeulen L . The landscape of cell-free mitochondrial DNA in liquid biopsy for cancer detection. Genome Biol. (2023) 24:229. doi: 10.1186/s13059-023-03074-w, PMID: 37828498 PMC10571306

[129] Mekata K Kyo M Tan M Shime N Hirohashi N . Molecular endotypes in sepsis: integration of multicohort transcriptomics based on RNA sequencing. J Intensive Care. (2025) 13:30. doi: 10.1186/s40560-025-00802-1, PMID: 40448231 PMC12123803

[130] Scicluna BP Cano-Gamez K Burnham KL Davenport EE Moore AR Khan S . A consensus blood transcriptomic framework for sepsis. Nat Med. (2025) 31:4119–30. doi: 10.1038/s41591-025-03964-5, PMID: 41028542 PMC12705454

[131] Scicluna BP van Vught LA Zwinderman AH Wiewel MA Davenport EE Burnham KL . Classification of patients with sepsis according to blood genomic endotype: a prospective cohort study. Lancet Respir Med. (2017) 5:816–26. doi: 10.1016/S2213-2600(17)30294-1, PMID: 28864056

[132] Fux E Lenski M Bendt AK Otvos JD Ivanisevic J De Bruyne S . A global perspective on the status of clinical metabolomics in laboratory medicine - a survey by the IFCC metabolomics working group. Clin Chem Lab Med. (2024) 62:1950–61. doi: 10.1515/cclm-2024-0550, PMID: 38915248

[133] Trongtrakul K Thonusin C Pothirat C Chattipakorn SC Chattipakorn N . Past Experiences for Future Applications of Metabolomics in Critically Ill Patients with Sepsis and Septic Shocks. Metabolites. (2021) 12:1. doi: 10.3390/metabo12010001, PMID: 35050123 PMC8779293

[134] Wang J Sun Y Teng S Li K . Prediction of sepsis mortality using metabolite biomarkers in the blood: a meta-analysis of death-related pathways and prospective validation. BMC Med. (2020) 18:83. doi: 10.1186/s12916-020-01546-5, PMID: 32290837 PMC7157979

[135] Ge J Deng Q Zhou R Hu Y Zhang X Zheng Z . Identification of key biomarkers and therapeutic targets in sepsis through coagulation-related gene expression and immune pathway analysis. Front Immunol. (2024) 15:1470842. doi: 10.3389/fimmu.2024.1470842, PMID: 39430765 PMC11486639

[136] George A Thomas J Welikumbura S Achhapalia Y . Lemierre's Syndrome: An Atypical Case of Fusobacterium necrophorum Bacteraemia in the Absence of Internal Jugular Vein Thrombosis. Cureus. (2024) 16:e66867. doi: 10.7759/cureus.66867, PMID: 39280464 PMC11397421

[137] George A . Lemierre's Syndrome: An Atypical Case of Fusobacterium necrophorum Bacteraemia in the Absence of Internal Jugular Vein Thrombosis. Cureus. (2024) 16:e66867. doi: 10.7759/cureus.66867, PMID: 39280464 PMC11397421

[138] Douglas IS Alapat PM Corl KA Exline MC Forni LG Holder AL . Fluid Response Evaluation in Sepsis Hypotension and Shock: A Randomized Clinical Trial. Chest. (2020) 158:1431–45. doi: 10.1016/j.chest.2020.04.025, PMID: 32353418 PMC9490557

[139] Fowler MJ Belay ES Hughes AJ Devine DK Chiu YF Carli AV . Moving Beyond Systemic Inflammatory Response Syndrome and Bacteremia: Are Modern Critical Care Calculators Useful in Predicting Debridement, Antibiotics, and Implant Retention Treatment Outcomes in Periprosthetic Joint Infection? J Arthroplasty. (2025) 40:1301–1307.e3. doi: 10.1016/j.arth.2024.10.127, PMID: 39491773

[140] Villanueva-Congote J Hinojosa-Gonzalez D Segall M Eisner BH . The relationship between neutrophil/lymphocyte ratio, platelet/neutrophil ratio, and risk of urosepsis in patients who present with ureteral stones and suspected urinary tract infection. World J Urol. (2024) 42:596. doi: 10.1007/s00345-024-05229-1, PMID: 39466513

[141] Chamani A Mashhadi F Khademi G Nematy M Emadzadeh M Sezavar M . Investigating the effect of synbiotic supplementation on inflammatory indices in critically ill septic children: a protocol study for randomized control trial. Trials. (2024) 25:712. doi: 10.1186/s13063-024-08514-x, PMID: 39443948 PMC11515531

[142] You R Quan X Xia P Zhang C Liu A Liu H . A promising application of kidney-specific cell-free DNA methylation markers in real-time monitoring sepsis-induced acute kidney injury. Epigenetics. (2024) 19:2408146. doi: 10.1080/15592294.2024.2408146, PMID: 39370847 PMC11459754

[143] Miller J Grahf D Nassereddine H Nehme J Rammal JA Ross J . Cross-Sectional Study of Thiamine Deficiency and Its Associated Risks in Emergency Care. West J Emerg Med. (2024) 25:675–9. doi: 10.5811/westjem.18472, PMID: 39319797 PMC11418880

[144] Miller J . Cross-Sectional Study of Thiamine Deficiency and Its Associated Risks in Emergency Care. West J Emerg Med. (2024) 25:675–9. doi: 10.5811/WESTJEM.18472, PMID: 39319797 PMC11418880

[145] Yan Y Zhu Z Ding H Zhu X Zhang J Fu C . Dexmedetomidine Alleviates Ferroptosis Induced by Sepsis-Induced Renal Injury by Activating Keap1-Nrf2 Signaling Pathway. Clin Lab. (2024) 70. doi: 10.7754/Clin.Lab.2024.240539, PMID: 39506587

[146] Priputnevich TV Gordeev AB Shabanova NE Denisov P Trofimov DY Balashova EN . The underestimated role of major skin commensal Malassezia furfur in the development of neonatal invasive fungal infections. Heliyon. (2024) 10:e38767. doi: 10.1016/j.heliyon.2024.e38767, PMID: 39502221 PMC11536008

[147] de Oliveira Veloso Rezende J Batista M Machado KC Bandini TB de Menezes IAC do Carmo De Stefani F . A dataset for developing proteomic tools for pathogen detection via differential cell lysis of whole blood samples. Sci Data. (2024) 11:1105. doi: 10.1038/s41597-024-03834-8, PMID: 39384817 PMC11464718

[148] Griffin K Miller L Yang Y Sharp E Young L Garcia L . Affinity-based 3D-printed microfluidic chip for clinical sepsis detection with CD69, CD64, and CD25. J Pharm BioMed Anal. (2025) 252:116500. doi: 10.1016/j.jpba.2024.116500, PMID: 39383543

[149] Rahman MS Islam KR Prithula J Kumar J Mahmud M Alam MF . Machine learning-based prognostic model for 30-day mortality prediction in Sepsis-3. BMC Med Inform Decis Mak. (2024) 24:249. doi: 10.1186/s12911-024-02655-4, PMID: 39251962 PMC11382400

[150] Liu F Yao J Liu C Shou S . Construction and validation of machine learning models for sepsis prediction in patients with acute pancreatitis. BMC Surg. (2023) 23:267. doi: 10.1186/s12893-023-02151-y, PMID: 37658375 PMC10474758

[151] Li L Ren J Fang Q Yu L Wang J . A predictive model for the classification of emergency intensive care unit patients with systemic inflammatory response syndrome based on a similarity network fusion algorithm. Neurosci Lett. (2023) 28:137538. doi: 10.1016/j.neulet.2023.137538, PMID: 39492503

[152] Li C Wang F Li W Sun G Yang D Yang T . The diagnostic value of metagenomic next-generation sequencing in critically ill patients with sepsis: A retrospective cohort study. Med (Baltimore). (2024) 103:e39987. doi: 10.1097/MD.0000000000039987, PMID: 39465842 PMC11479415

[153] Zheng X Meng D Chen D Wong WK To KH Zhu L . scCaT: An explainable capsulating architecture for sepsis diagnosis transferring from single-cell RNA sequencing. PloS Comput Biol. (2024) 20:e1012083. doi: 10.1371/journal.pcbi.1012083, PMID: 39432561 PMC11527285

[154] Lazzarino R Borek AJ Honeyford K Welch J Brent AJ Kinderlerer A . Views and Uses of Sepsis Digital Alerts in National Health Service Trusts in England: Qualitative Study With Health Care Professionals. JMIR Hum Factors. (2024) 11:e56949. doi: 10.2196/56949, PMID: 39405513 PMC11522658

[155] Becker JU Theodosis C Jacob ST Wira CR Groce NE . Surviving sepsis in low-income and middle-income countries: new directions for care and research. Lancet Infect Dis. (2009) 9:577–82. doi: 10.1016/S1473-3099(09)70135-5, PMID: 19695494

[156] Liu Y Zheng J Zhang D Jing L . Neutrophil-lymphocyte ratio and plasma lactate predict 28-day mortality in patients with sepsis. J Clin Lab Anal. (2019) 33:e22942. doi: 10.1002/jcla.22942, PMID: 31265174 PMC6757133

[157] Wang G Mivefroshan A Yaghoobpoor S Khanzadeh S Siri G Rahmani F . Prognostic Value of Platelet to Lymphocyte Ratio in Sepsis: A Systematic Review and Meta-analysis. BioMed Res Int. (2022) 2022:9056363. doi: 10.1155/2022/9056363, PMID: 35707370 PMC9192240

[158] Hernández G Ospina-Tascón GA Damiani LP Estenssoro E Dubin A Hurtado J . Effect of a Resuscitation Strategy Targeting Peripheral Perfusion Status vs Serum Lactate Levels on 28-Day Mortality Among Patients With Septic Shock: The ANDROMEDA-SHOCK Randomized Clinical Trial. JAMA. (2019) 321:654–64. doi: 10.1001/jama.2019.0071, PMID: 30772908 PMC6439620

[159] Vincent JL Quintairos E Silva A Couto L Jr Taccone FS . The value of blood lactate kinetics in critically ill patients: a systematic review. Crit Care. (2016) 20:257. doi: 10.1186/s13054-016-1403-5, PMID: 27520452 PMC4983759

[160] Moore CC Jacob ST Pinkerton R Meya DB Mayanja-Kizza H Reynolds SJ . Point-of-care lactate testing predicts mortality of severe sepsis in a predominantly HIV type 1-infected patient population in Uganda. Clin Infect Dis. (2008) 46:215–22. doi: 10.1086/524665, PMID: 18171253

[161] Li G Yang Z Yang C Xie Y Gong S Lv S . Single-cell RNA sequencing reveals cell-cell communication and potential biomarker in sepsis and septic shock patients. Int Immunopharmacol. (2024) 132:111938. doi: 10.1016/j.intimp.2024.111938, PMID: 38593502

[162] Xie L Zhao M Zong L Yue Y . Propofol Ameliorates Sepsis-Induced Myocardial Dysfunction via Anti-Apoptotic, Anti-Oxidative Properties, and mTOR Signaling. Discovery Med. (2024) 36:2088–97. doi: 10.24976/Discov.Med.202436189.193, PMID: 39463229

[163] Chen M Gao K Guo Z Feng L Feng Y Fu J . The Circulating CDC42 Expression in Sepsis: Relation to Disease Susceptibility, Inflammation, Multiple Organ Dysfunctions and Mortality Risk. Ann Clin Lab Sci. (2024) 54:525–32. 39293840

[164] Chen M . The Circulating CDC42 Expression in Sepsis: Relation to Disease Susceptibility, Inflammation, Multiple Organ Dysfunctions and Mortality Risk. Ann Clin Lab Sci. (2024) 54:525–32. 39293840

[165] Khanum I Kanwar D Habib K Mubarak F . Skull base osteomyelitis (SBO): a dreaded clinical entity. J Pak Med Assoc. (2024) 74:1767–72. doi: 10.47391/JPMA.9604, PMID: 39407368

[166] Qian H Shang W Zhang S Pan X Huang S Li H . The Circulating CDC42 Expression in Sepsis: Relation to Disease Susceptibility, Inflammation, Mult Trends and predictions of maternal sepsis and other maternal infections among women of childbearing age: a systematic analysis for the global burden of disease study 2019. Front Public Health. (2024) 12:1428271. doi: 10.3389/fpubh.2024.1428271, PMID: 39507668 PMC11538001

[167] Chen Z Lin B Yao X Fang Y Liu J Song K . OAS3 Deubiquitination Due to E3 Ligase TRIM21 Downregulation Promotes Epithelial Cell Apoptosis and Drives Sepsis-induced Acute Lung Injury. Int J Biol Sci. (2024) 20:5594–607. doi: 10.7150/ijbs.96089, PMID: 39494334 PMC11528449

[168] Garcia Lopez A Schäuble S Sae-Ong T Seelbinder B Bauer M Giamarellos-Bourboulis EJ . Risk assessment with gene expression markers in sepsis development. Cell Rep Med. (2024) :101712. doi: 10.1016/j.xcrm.2024.101712, PMID: 39232497 PMC11528229

[169] Garcia Lopez A . Risk assessment with gene expression markers in sepsis development. Cell Rep Med. (2024) 5:101712. doi: 10.1016/j.xcrm.2024.101712, PMID: 39232497 PMC11528229

[170] Dias FS . Sepsis definitions. Rev Bras Ter Intensiva. (2017) 29:520–1. doi: 10.5935/0103-507X.20170074, PMID: 29340542 PMC5764565

[171] Machado FR Assunção MS Cavalcanti AB Japiassú AM Azevedo LC Oliveira MC . Getting a consensus: advantages and disadvantages of Sepsis 3 in the context of middle-income settings. Rev Bras Ter Intensiva. (2016) 28:361–5. doi: 10.5935/0103-507X.20160068, PMID: 28099632 PMC5225908

[172] Waked R Coats L Rosato A Yen CF Wood E Diekema DJ . Clinical outcome of combination of vancomycin and ceftaroline versus vancomycin monotherapy for treatment of methicillin resistant Staphylococcus aureus bloodstream infection. BMC Infect Dis. (2024) 24:1212. doi: 10.1186/s12879-024-10107-7, PMID: 39468491 PMC11514869

[173] But Š Celar B Fister P . Tackling Neonatal Sepsis-Can It Be Predicted? Int J Environ Res Public Health. (2023) 20:3644. doi: 10.3390/ijerph20043644, PMID: 36834338 PMC9959311

[174] Wang Z Wang Y Dong C Miao K Jiang B Zhou D . Po-Ge-Jiu-Xin decoction alleviate sepsis-induced cardiomyopathy via regulating phosphatase and tensin homolog-induced putative kinase 1 /parkin-mediated mitophagy. J Ethnopharmacol. (2025) 337:118952. doi: 10.1016/j.jep.2024.118952, PMID: 39426573

[175] Mosly MM Mousli HM Ahmed IMM Abdou MIA . Cost-effectiveness of Procalcitonin (PCT) guidance for antibiotics management of adult sepsis patients in the Egyptian context. BMC Health Serv Res. (2024) 24:1249. doi: 10.1186/s12913-024-11675-9, PMID: 39420348 PMC11484283

[176] Özer A Tak S Demirtaş H Yıldırım AK Şimşek E Oktar GL . The Role of Monocyte Distribution Width in the Early Prediction of Sepsis in Patients Undergoing Cardiovascular Surgery: A Cross-Sectional Study. Medicina (Kaunas). (2024) 60:1558. doi: 10.3390/medicina60091558, PMID: 39336599 PMC11434002

[177] Gomez DE Kamr A Gilsenan WF Burns TA Mudge MC Hostnik LD . Endothelial glycocalyx degradation in critically ill foals. J Vet Intern Med. (2024) 38:2748–57. doi: 10.1111/jvim.17196, PMID: 39275920 PMC11423458

[178] Gomez DE . Endothelial glycocalyx degradation in critically ill foals. J Vet Intern Med. (2024) 38:2748–57. doi: 10.1111/jvim.17196, PMID: 39275920 PMC11423458

[179] Cao Q Li J Chen M . Bioinformatics analysis of neutrophil-associated hub genes and ceRNA network construction in septic cardiomyopathy. Aging (Albany NY). (2024) 16:12833–49. doi: 10.18632/aging.206092, PMID: 39216003 PMC11501391

[180] Mittal A Kumar D Panwar VK Ranjan R Navriya SC Upadhyaya A . Scoring System to Personalize Management of Emphysematous Pyelonephritis. Urol Res Pract. (2024) 50:193–7. doi: 10.5152/tud.2024.23165, PMID: 39499022 PMC11562922

[181] Ouyang X Fu M Li J Gao J Xu L Pei Y . Risk factors for occurrence and death of sepsis-associated acute kidney injury in children with sepsis. Int Immunopharmacol. (2024) 143:113551. doi: 10.1016/j.intimp.2024.113551, PMID: 39488919

[182] Evangelatos N Satyamoorthy K Levidou G Bauer P Brand H Kouskouti C . Multi-Omics Research Trends in Sepsis: A Bibliometric, Comparative Analysis Between the United States, the European Union 28 Member States, and China. OMICS. (2018) 22:190–7. doi: 10.1089/omi.2017.0192, PMID: 29649387

[183] Zhang TN Wen R Yang YH Yang N Liu CF . Integration of transcriptomic, proteomic, and metabolomic data to identify lncRNA rPvt1 associations in lipopolysaccharide-treated H9C2 cardiomyocytes. Front Genet. (2023) 14:1278830. doi: 10.3389/fgene.2023.1278830, PMID: 38094756 PMC10718647

[184] Zhang TN . Integration of transcriptomic, proteomic, and metabolomic data to identify lncRNA rPvt1 associations in lipopolysaccharide-treated H9C2 cardiomyocytes. Front Genet. (2023) 14:1278830. doi: 10.3389/fgene.2023.1278830, PMID: 38094756 PMC10718647

[185] Schuurman AR Reijnders TDY Kullberg RFJ Butler JM van der Poll T Wiersinga WJ . Sepsis: deriving biological meaning and clinical applications from high-dimensional data. Intensive Care Med Exp. (2021) 9:27. doi: 10.1186/s40635-021-00383-x, PMID: 33961170 PMC8105470

[186] Schuurman AR . Sepsis: deriving biological meaning and clinical applications from high-dimensional data. Intensive Care Med Exp. (2021) 9:27. doi: 10.1186/s40635-021-00383-x, PMID: 33961170 PMC8105470

[187] Zhang C Singla RK Tang M Shen B . Natural products act as game-changer potentially in treatment and management of sepsis-mediated inflammation: A clinical perspective. Phytomedicine. (2024) 130:155710. doi: 10.1016/j.phymed.2024.155710, PMID: 38759311

[188] Kim MJ Choi EJ Choi EJ . Evolving Paradigms in Sepsis Management: A Narrative Review. Cells. (2024) 13:1172. doi: 10.3390/cells13141172, PMID: 39056754 PMC11274781

[189] Behroozizad N Mahmoodpoor A Shadvar K Ardebil RA Pahnvar AJ Sohrabifar N . Evaluation of circulating levels of miR-135a and miR-193 in patients with sepsis. Mol Biol Rep. (2024) 51:282. doi: 10.1007/s11033-024-09225-x, PMID: 38324210

[190] Ruiz-Rodriguez JC Plata-Menchaca EP Chiscano-Camón L Ruiz-Sanmartin A Pérez-Carrasco M Palmada C . Precision medicine in sepsis and septic shock: From omics to clinical tools. World J Crit Care Med. (2022) 11:1–21. doi: 10.5492/wjccm.v11.i1.1, PMID: 35433311 PMC8788206

[191] Srdić T Đurašević S Lakić I Ružičić A Vujović P Jevđović T . From Molecular Mechanisms to Clinical Therapy: Understanding Sepsis-Induced Multiple Organ Dysfunction. Int J Mol Sci. (2024) 25:7770. doi: 10.3390/ijms25147770, PMID: 39063011 PMC11277140

[192] Miao H Chen S Ding R . Evaluation of the Molecular Mechanisms of Sepsis Using Proteomics. Front Immunol. (2021) 12:733537. doi: 10.3389/fimmu.2021.733537, PMID: 34745104 PMC8566982

